# Supplementary material for: Switching molecular recognition selectivities by temperature in a diffusion-regulatory porous material
Source: Nat Commun. 2024 Jan 2;15:144. doi: 10.1038/s41467-023-44424-3 (PMC10761840; doi:10.1038/s41467-023-44424-3)
Supplement: Supplementary file 1 — Supplementary Information [file 41467_2023_44424_MOESM1_ESM.pdf]

Supplementary information

Switching molecular recognition selectivities by temperature in a diffusion-regulatory porous material

Yan Su[1], Ken-ichi Otake[3], Jia-Jia Zheng[4], Hong Xu[5], Qing Wang[6], Haiming Liu[6], Fei Huang[7], Ping Wang[2], Susumu Kitagawa[3] & Cheng Gu[1,2]

*Corresponding to kitagawa@icems.kyoto-u.ac.jp; gucheng@scu.edu.cn

This PDF file includes:

Table of Contents
Supplementary Materials and Methods
Supplementary Figures 1 to 51
Supplementary Tables 1 to 7
Supplementary References (1−23)

Table of Contents

1. Supplementary Materials and Methods..P3−P7
Section 1: Materials and PCP synthesis...P3
Section 2: Instruments and characterizations..P3−P5
Section 3: Theoretical calculation..P5−P6
Section 4: Gas separation..P6−P7

2. Supplementary Figures 1 to 51..P8−P59
Section 1: Calculated flip-flop energy of the ligand...P8
Section 2: Morphology, infrared spectra, and crystal structures of FDCs..........................P9−P14
Section 3: ^{1}H NMR, TG, and PXRD of as-synthesized and activated FDCs......................P15−P17
Section 4: Gas-sorption behaviors of FDC–3a..P18−P22
Section 5: IAST selectivities of FDC–3a..P23
Section 6: Global pressure–diffusion-rate–adsorption amount curves for FDC–3a..................P24
Section 7: PXRD and flip-flop mechanism...P25−P29
Section 8: Solid-state NMR..P30−P33
Section 9: Theoretical calculation of the diffusion energies and rates....................................P34−P35
Section 10: Gas separation experiments for FDC–3a...P36−P59

3. Supplementary Tables 1 to 7..P60−P66
Section 1: Crystallographic data for as-synthesized and activated FDCs..............................P60−P61
Section 2: Structures and physicochemical properties of CO_2 and C_2H_2..................................P62
Section 3: Calculation of IAST selectivity..P63−P64
Section 4: Solid-state NMR..P65
Section 5: Theoretical calculations...P66

4. Supplementary References (1−23)..P67−P68

Supplementary Materials and Methods

Section 1: Materials and PCP synthesis

Materials

5'-(5,5-Dioxido-10H-phenothiazin-10-yl)-[1,1':3',1''-terphenyl]-3,3''-dicarboxylic acid (OPTz-t3da, 98%, Jilin Chinese Academy of Sciences-Yanshen Technology Co., Ltd.), anhydrous *N,N*-dimethylacetamide (99.5%, Energy Chemical), anhydrous methanol (99.5%, Energy Chemical), 12 M DCl (Aldrich), Zinc nitrate (99.0 %, Aldrich), deuterated solvents for nuclear magnetic resonance (NMR) spectroscopy (Cambridge Isotope Laboratories) were purchased and used without further purification.

N_2 (99.9999%), CO (99.9999%), O_2 (99.9999%), Ar (99.9999%), C_2H_4 (99.9999%), C_2H_6 (99.9999%), CO_2 (99.9999%), C_2H_2 (99.9999%), and He (99.9999%) were purchased from TAIYO NIPPON SANSO Company (Japan).

Synthesis of **FDC–3**

Firstly, 50 mg (0.09 mmol) **OPTz-t3da** was dissolved in 2 mL DMA at room temperature. A methanol solution (8 mL) of $Zn(NO_3)_2 \cdot 6H_2O$ (54 mg, 0.18 mmol) was added to the above solution. Then the mixture was heated at 80 °C for 72 h. **FDC–3** was obtained as colorless block crystals with sizes up to several hundreds of micrometers (37 mg, yield = 43%). The crystals were filtered, washed with DMA (10 mL, 3 times) and methanol (10 mL, 3 times), and dried in air. The as-synthesized **FDC–3** was characterized by infrared spectra (Supplementary Fig. 3). The adsorption peak of the stretching vibration of the C=O double bond shifted to a low wavenumber, indicative of the coordination bond formation in **FDC–3**.

Solvent exchange and activation of **FDC–3**

To measure the adsorption property of **FDC–3**, we exchanged the guest and coordination solvents (DMA) with methanol by soaking **FDC–3** in methanol at 60 °C for 7 days. Every 24 h the methanol was replaced by a new one. After the solvent exchange, the exchanged **FDC–3** was dried under vacuum at 60 °C for 3 h. ^{1}H NMR confirmed that the DMA in the exchanged **FDC–3** was exchanged by methanol (Supplementary Fig. 8).

TG curve showed that the framework of the exchanged **FDC–3** was thermally stable until 391 °C, whereas below 60 °C the exchanged **FDC–3** lost the methanol molecules (Supplementary Fig. 9). Thus, we activated the exchanged **FDC–3** at 120 °C for 11 h to afford **FDC–3a**; this temperature ensured the complete removal of the solvents meanwhile excluding the possibility of framework decomposition.

Section 2: Instruments and characterizations

General instrumental analysis

^{1}H NMR spectra were recorded at 25 °C on a Brucker models Ultrashild 500 Plus NMR spectrometer operating at 500 MHz, where chemical shifts (δ in ppm) were determined with respect to tetramethylsilane (TMS) as an internal reference. IR spectra were obtained with a Thermo Scientific Nicolet Summit FT-IR equipped with a diamond ATR accessory under an ambient atmosphere. Thermogravimetric analysis (TGA) was performed on a Rigaku Thermo plus EVO2 under a nitrogen atmosphere with a temperature ramp of 5 °C min^{-1}.

X-ray diffraction

Single-crystal X-ray diffraction (SCXRD) for **FDC–3** was performed on a Rigaku XtaLAB P200 diffractometer equipped with a Dectoris PILATUS 200 K detector, using a VariMax Mo Optic with Mo−Kα radiation (λ = 0.71075 Å). The structure was solved using direct methods and refined by full-matrix least-squares cycles in SHELX 2014/7[1]. All non-hydrogen atoms were refined using anisotropic thermal parameters.

Powder X-ray diffraction (PXRD) measurements were performed on a Rigaku SmartLab X-ray diffractometer using Cu−Kα radiation (λ = 1.54178 Å) in the 2θ range of 4−40° with a scanning rate of 5° min^{-1}. Variable-temperature synchrotron powder X-ray diffraction measurements were conducted using high-resolution one-dimensional semiconductor (MYTHEN) detectors (Dectris, Switzerland) installed in the BL02B2 beamline of SPring-8 (λ = 0.80000 Å) at varied temperatures[2,3]. The crystalline powder of **FDC–3a** in a borosilicate glass capillary (0.5 mm inside diameter) was activated at 393 K under vacuum for 1 h. The measurement temperature was changed from 380 to 100 K at the cooling rate of 10 K min^{-1} with nitrogen flow.

Continuous rotation electron diffraction (cRED)

The crystal powder was drop-casted onto a copper grid (R1.2/1.3, QUANTIFOIL), and the grid was plunged into liquid nitrogen rapidly. The grid was then transferred to the Fischone 2550 cryo holder and TEM at liquid nitrogen temperature (100 K). The cRED data were collected on a JEOL 2100-plus TEM equipped with MerlinEM direct electron detector under 200 kV acceleration voltage and installed with Heimdall data collection software (software developed by the ReadCrystal Tech Co.). The data were visualized with the program REDp (Wan et al., 2013) and processed using XDS (Kabsch, 2010) with the aid of Coeus (software developed by the ReadCrystal Tech Co.)[4].

Crystal structure models were built directly using the Shelxt package, and more accurate structure models were obtained by the ShelxL refinement.

Gas sorption

Gas sorption measurements were performed on BELSORP-max and BELSORP-18PLUS (MicrotracBEL, Japan, Corp.) automated volumetric sorption analyzers, equipped with cryostatic temperature controllers.

Calculation of the IAST selectivity

To calculate the IAST selectivity, the singe-component CO_2 and C_2H_2 adsorption isotherms for the adsorbate at different temperatures were first fitted to the dual-site Langmuir-Freundlich equation (equation 1):

$$q = n_{m1}\frac{b_1P^{(1/t_1)}}{1+b_1P^{(1/t_1)}} + n_{m2}\frac{b_2P^{(1/t_2)}}{1+b_2P^{(1/t_2)}} \tag{1}$$

where q is the amount adsorbed per mass of material (mmol g^{-1}); P is the total pressure (kPa) of the bulk gas at equilibrium with the adsorbed phase; n_{m1} and n_{m2} are the saturation uptakes (mmol g^{-1}) for sites 1 and 2, respectively; b_1 and b_2 are the affinity coefficients (kPa^{-1}) for sites 1 and 2, respectively; t_1 and t_2 represent the deviations from the ideal homogeneous surface (dimensionless) for sites 1 and 2, respectively.

Then, the IAST selectivity (S) is defined as:

$$S = \frac{q_1 / q_2}{p_1 / p_2} \tag{2}$$

where q_1 and q_2 are the molar loadings in the adsorbed phase in equilibrium with the bulk gas phase with partial pressures p_1 and p_2 (Supplementary Tables 4 and 5).

Quantification of the diffusion rate

The diffusion rate was measured on a BEL-18 (BEL Japan, Inc.) automated volumetric sorption analyzer and was fitted automatically with BEL-Dyna software according to the Crank theory[5] described as follows:

Adsorption rate equation in consideration of in-particle diffusion (assuming spherical particle):

$$\frac{\partial q}{\partial t} = D_s\left(\frac{\partial^2 q}{\partial r^2} + \frac{2}{r} \times \frac{\partial q}{\partial r}\right) \tag{3}$$

When boundary condition $r = R$, $q = q_{0n}$

Linear equilibrium equation:

$$q^* = H \times P \tag{4}$$

Batch adsorption operating equation:

$$W(q - q_{0n}) = V(P_{0n} - P) \tag{5}$$

where

D_s: surface diffusion coefficient [cm^2 s^{-1}]
H: equilibrium constant [cm^3 g^{-1}]
P: pressure [Pa]
P_0: initial pressure [Pa]
P_e: equilibrium pressure [Pa]
q: adsorbed amount [cm^3 g^{-1}]
q_0: initial adsorption amount [cm^3 g^{-1}]
R: radius of particle [cm]
t: time [s]
V: gas phase volume [cm^3]
W: adsorbent amount [g]

By solving simultaneous equations of the above (3), (4), and (5), the following solution is obtained:

$$\frac{P}{P_{0n}} = 1 - \frac{1}{\alpha + 1}\left\{1 - \sum_{n=1}^{\infty} \frac{6\alpha(\alpha + 1)\exp(-q_n^2 \tau_s)}{9 + 9\alpha + q_n^2 \alpha^2}\right\} \tag{6}$$

Equation (4) is called the Crank equation.

$$\frac{P - P_{en}}{P_{0n} - P_{en}} = 1 - \left(\frac{P_{0n} - P_{en-1}}{P_{0n} - P_{en}}\right)\left(\frac{1}{\alpha + 1}\right)\left\{1 - \sum_{n=1}^{\infty} \frac{6\alpha(\alpha + 1)\exp(-q_n^2 \tau_s)}{9 + 9\alpha + q_n^2 \alpha^2}\right\} \tag{7}$$

Providing that P = CRT, equation (5) is as follows:

$$\frac{C - C_{en}}{C_{0n} - C_{en}} = 1 - \left(\frac{C_{0n} - C_{en-1}}{C_{0n} - C_{en}}\right)\left(\frac{1}{\alpha + 1}\right)\left\{1 - \sum_{n=1}^{\infty} \frac{6\alpha(\alpha + 1)\exp(-q_n^2 \tau_s)}{9 + 9\alpha + q_n^2 \alpha^2}\right\} \tag{8}$$

where

$$\tau_s = \frac{D_s}{R^2} t\,,\ \alpha = \frac{V}{W \times H}$$

For the adsorption rate analysis program, equation (8) is used.

The diffusion rate was simultaneously measured with every plot in the adsorption curves and was quantified in the temperature range of 200 to 360 K.

Solid-state NMR (SSNMR)

All variable temperature (VT) solid-state ^{1}H magic angle spinning (MAS) and ^{1}H-^{13}C cross polarization (CP) MAS NMR experiments were performed between -60 and 140 °C on a Bruker

Avance III HD 400WB (9.4 T) spectrometer, operating at 400.2 MHz for ^{1}H and 100.6 MHz for ^{13}C, at a spinning speed (SS) of 14 kHz on a 4.0 mm CPMAS probe. Activated **FDC–3a** was packed into a zirconia rotor inside a glove box under nitrogen, and then exposed to oxygen for 30 min for O_2-enhanced CP experiments. O_2-enhanced CP spectra were acquired under dry air and nitrogen with a ^{1}H $\pi/2$ pulse of 3.571 μs, a contact time of 5 ms and a recycle time of 10-12 s. High-power ^{1}H decoupling was carried out using SPINAL64 with a typical field strength of 80 kHz. The ^{13}C chemical shifts were referenced externally to α-glycine carbonyl residual at 176.03 ppm. The actual sample temperature was calibrated using ^{207}Pb MAS NMR of solid lead nitrate under the same condition[6], and the actual temperature of RT (room temperature) was 45 °C. Linewidth ($\Delta_{1/2}$) as full-width at half-maximum was measured directly or by spectral deconvolution throughout this work. ACD NMR Workbook Suite was used for the calculation of solution ^{13}C NMR chemical shifts of the OPTz-t3da ligand.

Section 3: Theoretical calculation

Theoretical calculation

The crystalline structures of **FDC–3** and **FDC–3a** were calculated using density-functional theory (DFT)[7,8] implemented in the CASTEP[9] module of Materials Studio 7.0. The generalized gradient approximation (GGA) in the form of Perdew–Burke–Ernzerhof (PBE)[10] was selected as the exchange-correlation functional. Grimme dispersion correction[11,12] was employed in all calculations to describe van der Waals (vdW) interactions. The lattice lengths along with plane directions were optimized simultaneously with the geometry. A plane wave energy cutoff of 830 eV was used, and the convergence criteria for energy, force, stress, and displacement are 5×10^{-6} eV atom^{-1}, 0.01 eV Å^{-1}, 0.02 GPa, and 5×10^{-4} Å, respectively. The DFT calculated lattice parameters and the geometry of the framework are quite close to those obtained by single-crystal XRD measurement (Supplementary Table 2), indicating that the above calculation method could provide a good description of **FDC–3a** and the interactions of its pore walls.

To search the adsorption positions of guest molecules (CO_2 and C_2H_2), we carried out Metropolis Monte Carlo simulation[13], using Universal force field[14–16] to describe the Van der Waals interaction between guest molecules and **FDC–3a** pore walls. The low-energy adsorption conformations returned by the Monte Carlo search were further optimized using DFT with the same parameters as above. The cell parameters were kept the same as those of the activated phase because adsorptions of CO_2 and C_2H_2 did not change the structure of **FDC–3a** very much (Supplementary Figure S10). To reflect the deformation of **FDC–3a** upon interaction with guest molecules, the geometry of the framework of **FDC–3a** was optimized simultaneously with guest molecules. The energy barrier of guest molecule diffusion was calculated using the single-point energy of each loading position of the obtained guests-loading saturated **FDC–3a**. Due to the periodic structure of **FDC–3a**, the diffusion paths of both CO_2 and C_2H_2 are composed of 5 repeating molecules (Figure 3b,c); The molecules at the initial position are marked in red color, and the molecules at the other positions of the diffusion pathways are marked with orange, yellow, green, and cyan colors, respectively. All simulation works were performed using the computing resources at the National Supercomputing Center in Shenzhen.

The energy change for the flipping of the OPTz ring in the free OPTz-t3da ligand was calculated using Gaussian 16 program[17]. Geometry optimizations and vibrational frequency calculations were carried out using the DFT method. The dispersion-corrected[18] B3LYP functional (B3LYP-D3) with the 6-311G(d,p) basis sets for all atoms were used in these calculations. The flipping barrier was estimated by scanning the potential energy surface (PES), in which one

dihedral angle (C^1NSC^2, Supplementary Fig. 1) was changed from 120° to 200° with an interval of 5°.

Section 4: Gas separation

Gas separation experiments by temperature-programmed desorption (TPD) protocol

The TPD experiments on **FDC–3a** were carried out as the following steps using BEL-CAT II cooperated with a mass detector (MicrotracBEL, Japan, Corp.). All experimental steps were performed at atmospheric pressure. The steps below were carried out similarly to the steps reported previously[19,20].

1. Sample loading and activation:

The powder sample of **FDC–3a** (0.50 g) was filled in the cylindrical cell (8 mmϕ) and activated at 393 K under vacuum for 10 h. The sample cell was then placed on BEL-CAT II. The sample was then *in situ* activated by flowing He at a constant rate of 10 sccm for 1 h at 393 K.

2. Adsorption process:

The temperature was decreased from 393 to 240 K (or 320 K) at a rate of 10 K min^{-1}. Once the temperature reached 240 K (or 320 K), the mixed gases of CO_2 and C_2H_2 (volume ratios controlled by mass flow controllers, the total flow rate at 10 sccm) flowed at ambient pressure, and the temperature was kept at 240 K (or 320 K) for a certain period under flowing mixed gases.

3. Flowing away the non-adsorbed gases:

The remained gases in the cell and gas lines were flowed away by He with a flow rate of 10 sccm for 1 h at 240 K (or 320 K).

4. Release and detection of the adsorbed gases:

After flowing away the non-adsorbed gases, the temperature of the sample cell was increased to 393 K at a rate of 10 K min^{-1} for releasing the adsorbed gases, while the He flow was kept at a 10 sccm flow rate to carry the gases to the detector. The released gases were examined with a mass detector and the ratios between CO_2 and C_2H_2 were evaluated from the mass signal. The amounts of released CO_2 and C_2H_2 were calculated by integrating the area of the mass signal after the baseline subtraction.

The separation factor α is defined as:

$$\alpha = \frac{X_{CO_2}/Y_{CO_2}}{X_{C_2H_2}/Y_{C_2H_2}} = \frac{X_{CO_2}/X_{C_2H_2}}{Y_{CO_2}/Y_{C_2H_2}} \quad (9)$$

where:

X_{CO2} = the concentration of CO_2 in the adsorbed phase,

Y_{CO2} = the concentration of CO_2 in the feed gas,

X_{C2H2} = the concentration of C_2H_2 in the adsorbed phase,

Y_{C2H2} = the concentration of C_2H_2 in the feed gas.

Supplementary Figures
Section 1: Calculated flip-flop energies of the ligand

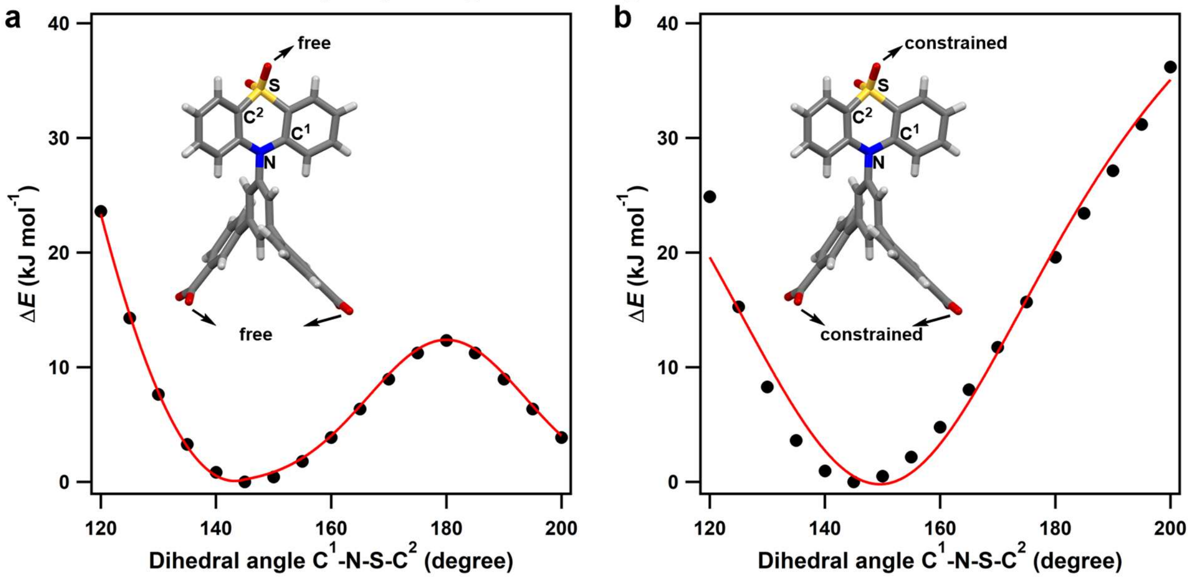

Supplementary Figure 1. (a) Potential energy surface for the flip-flop of OPTz ring in free ligand molecules. (b) Potential energy surface for the flip-flop of OPTz ring in oxygen atom-constrained ligand molecules to simulate the activated PCP. The energy change (ΔE) for the OPTz ring flipping is less than 25 kJ mol^{-1} with respect to a change of 25° in the dihedral angle. Therefore, the thermal flipping of the OPTz ring is feasible.

Section 2: Morphology, infrared spectra, and crystal structures of FDCs

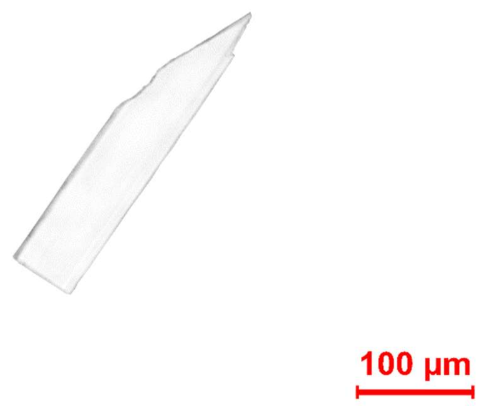

Supplementary Figure 2. Microscopy image of as-synthesized **FDC–3**.

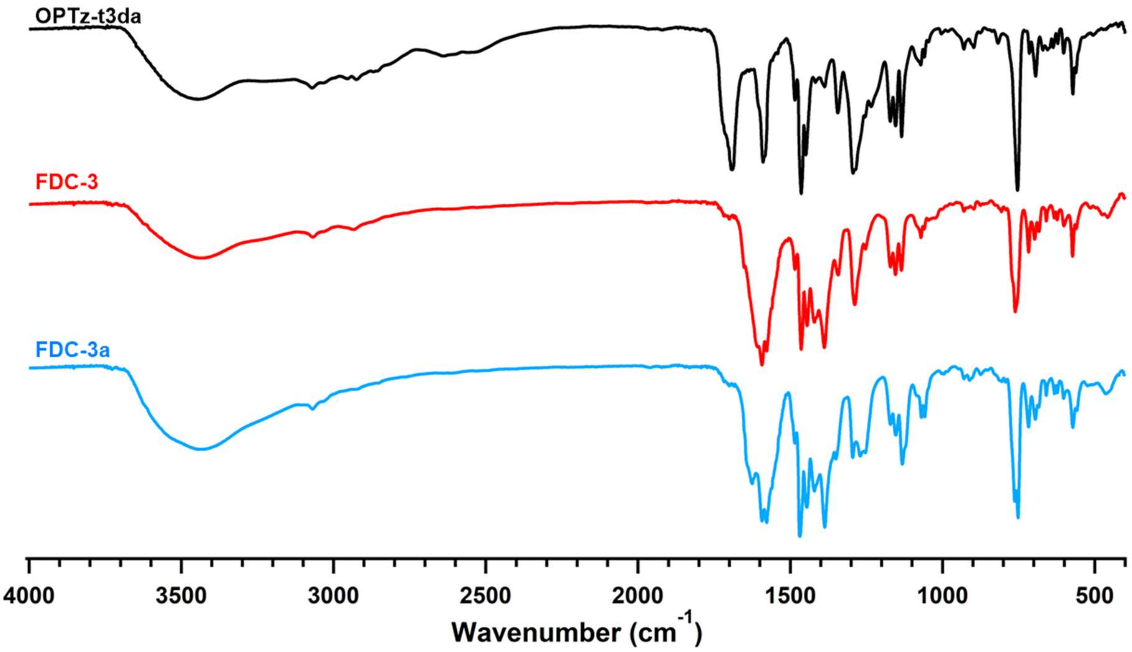

Supplementary Figure 3. Infrared spectra of the ligand, **FDC–3**, and **FDC–3a**.

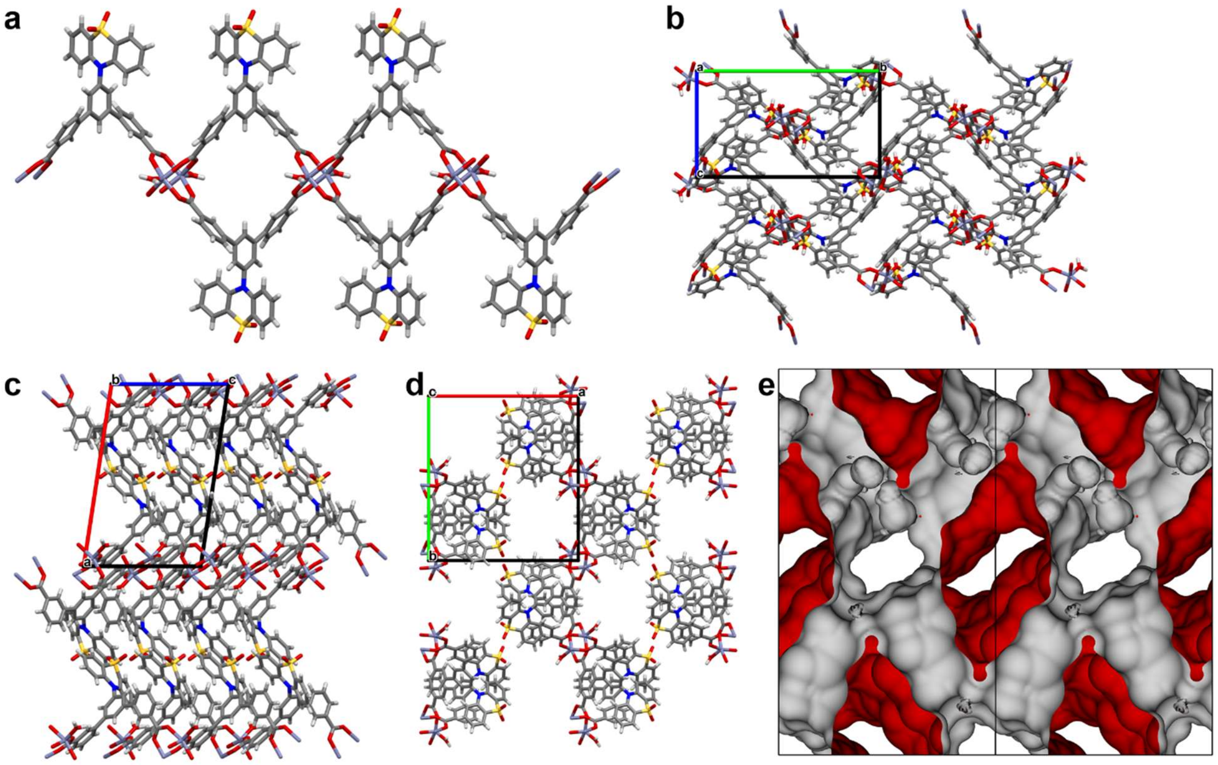

Supplementary Figure 4. Single-crystal structure of **FDC–3**. (a) Zn^{2+} paddle-wheel linked with OPTz-t3da ligands and the linear framework structure of **FDC–3**. For clarity, the guest solvents are omitted. (b), (c) and (d) represent the view of the crystal structure along the *a*-, *b*-, and *c*-axis, respectively. (e) Void structure of **FDC–3**. C: grey; N: blue; H: white; O: red; S: yellow; Zn: light purple. The inner and outer surfaces are in red and grey colors, respectively.

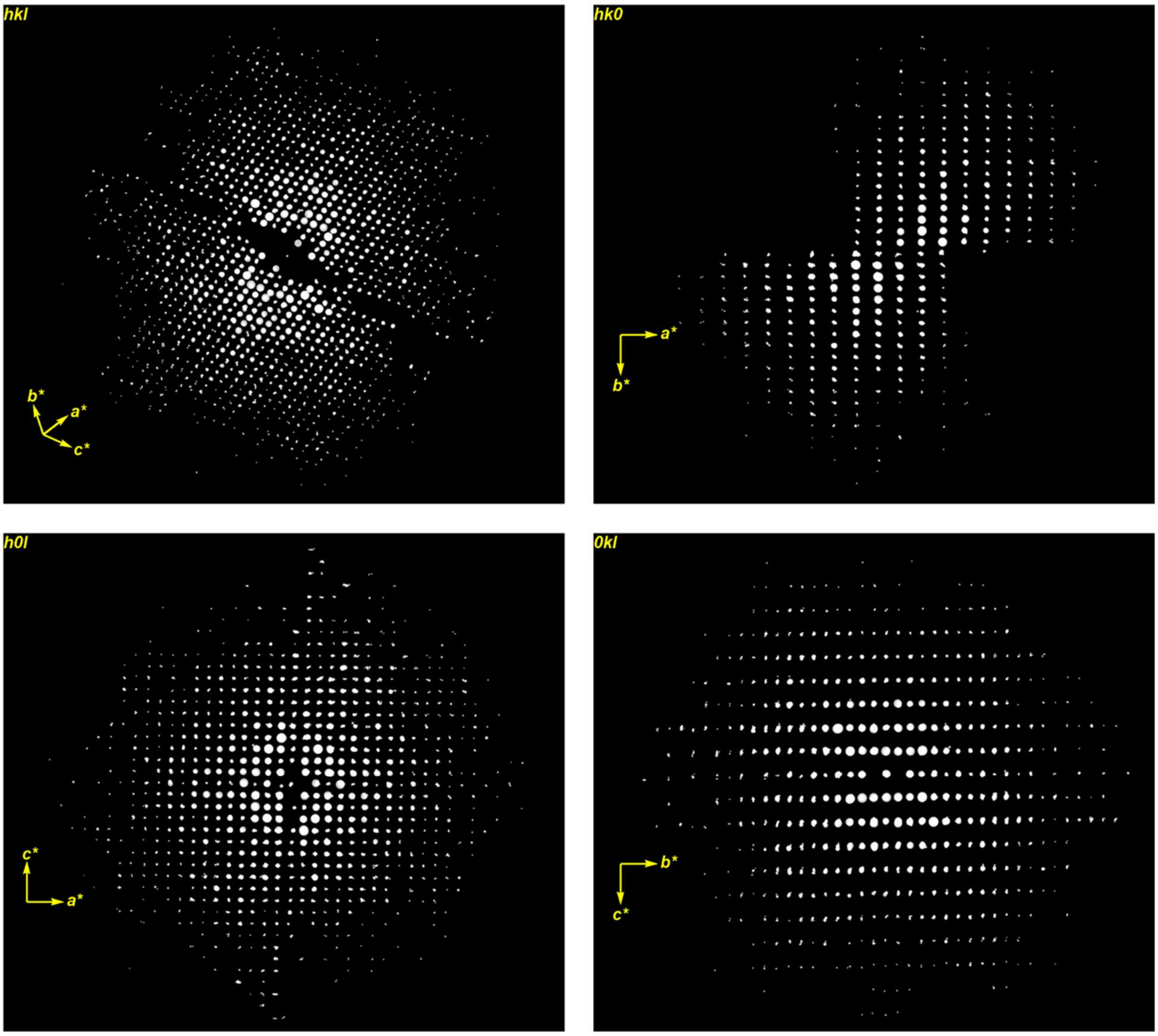

Supplementary Figure 5. The 3D reciprocal lattice of **FDC–3a** by the cRED technique.

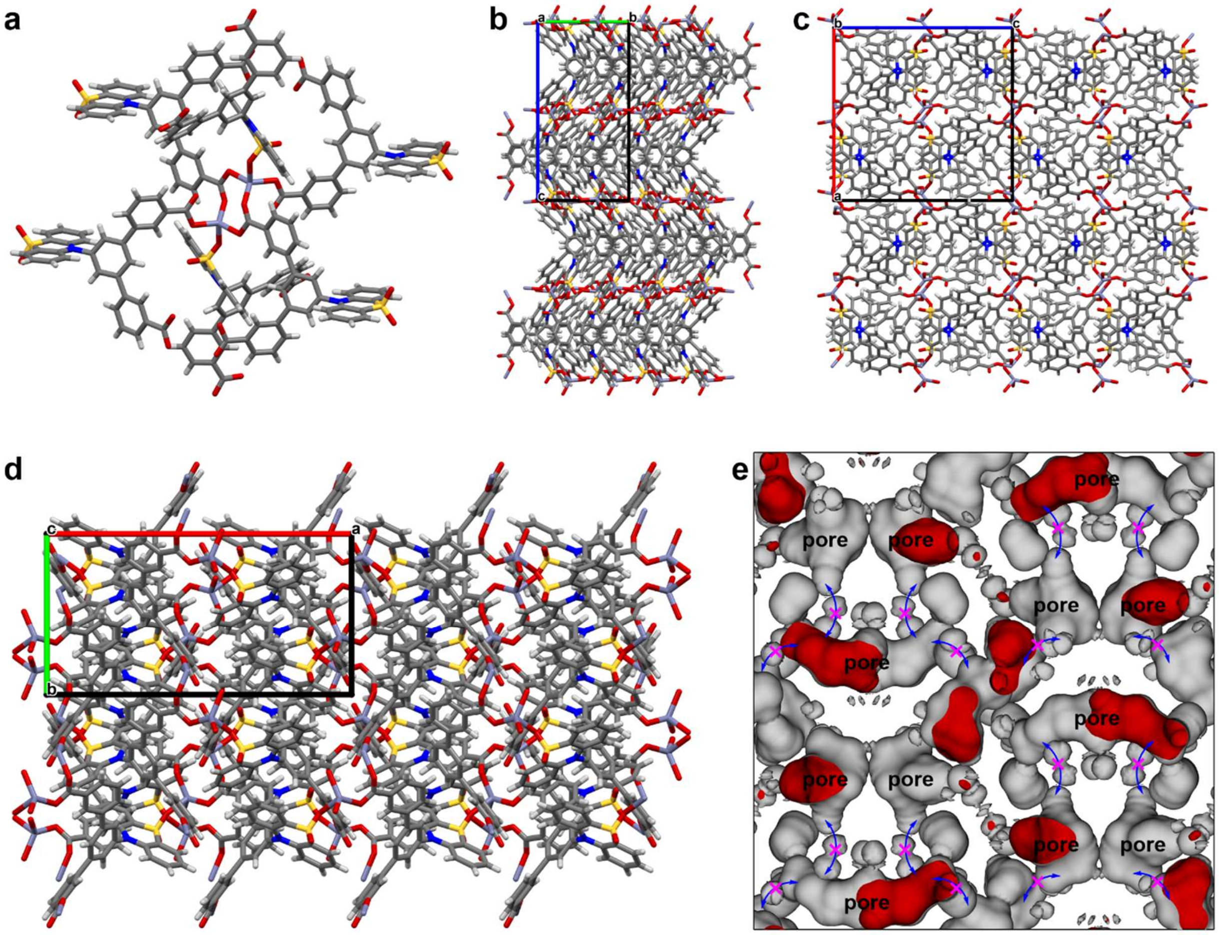

Supplementary Figure 6. Single-crystal structure of **FDC–3a**. (a) Zn^{2+} tetrahedron linked with OPTz-t3da ligands. (b), (c) and (d) represent the view of the crystal structure along the *a*-, *b*-, and *c*-axis, respectively. (e) Void structure of **FDC–3a**. C: grey; N: blue; H: white; O: red; S: yellow; Zn: light purple. The inner and outer surfaces are in red and grey colors, respectively. The blue arrows denote the diffusion windows that are closed at low temperatures to block the gas diffusion.

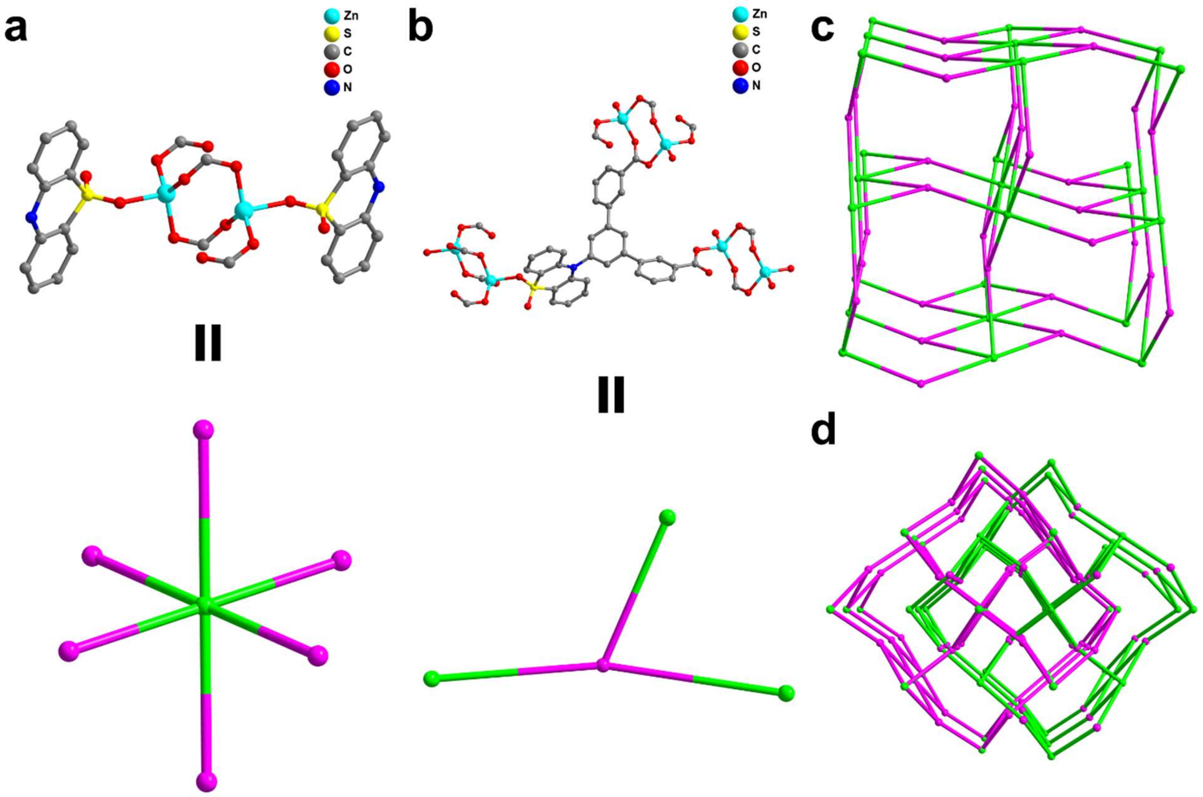

Supplementary Figure 7. Topological analysis for **FDC–3a**. (a) The Zn^{2+} dual-tetrahedron cluster possesses 6 coordination sites, which is simplified as a 6-connected node and is represented with green balls. For clarity, hydrogen atoms are omitted. (b) The ligand is linked with 3 Zn^{2+} clusters, which is simplified as a 3-connected node and is represented with purple balls. For clarity, hydrogen atoms are omitted. (c) The 3, 6-connected rutile (rtl) topology. (d) The two-fold interpenetrated, 3, 6-connected rutile (rtl) topology of **FDC–3a**.

Section 3: ^{1}H NMR, TG, and PXRD of as-synthesized and activated FDCs

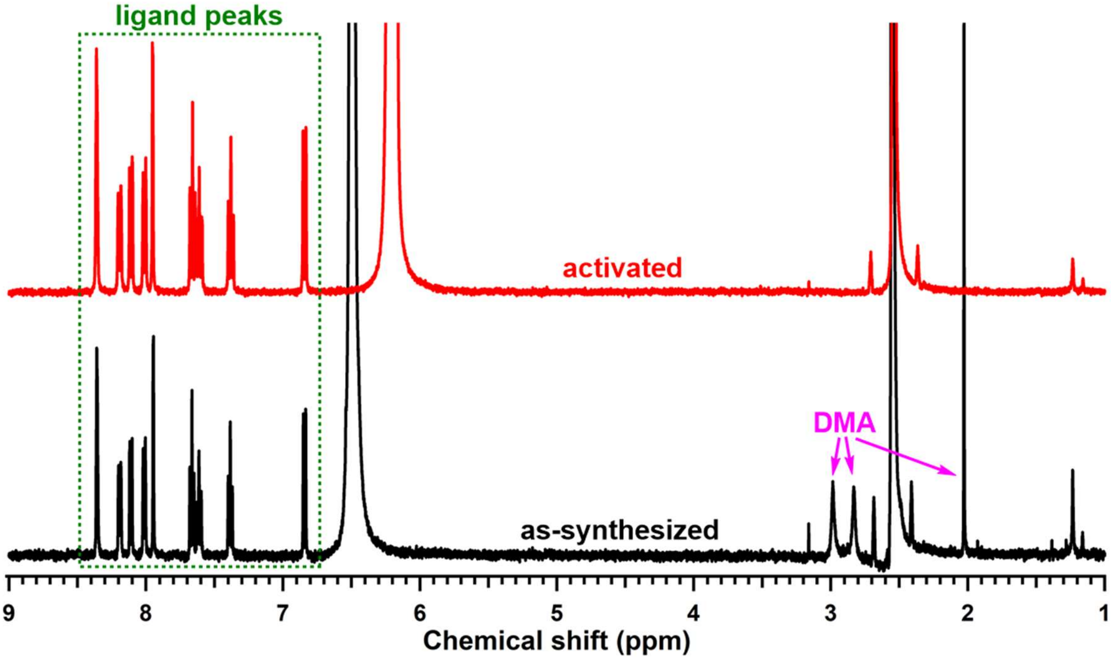

Supplementary Figure 8. ^{1}H NMR of the as-synthesized and activated **FDC–3** dissolved in DMSO-d_6 containing deuterium chloride. The PCP samples were digested by deuterium chloride (1 drop) in DMSO-d_6. The black and red curves represented the as-synthesized and activated PCPs, respectively. The peaks in the green box were the peaks of the OPTz-t3da ligand, whereas the peaks pointed by pink arrows were the peaks of DMA. The NMR clearly showed that the DMA molecules in **FDC–3** were exchanged by methanol.

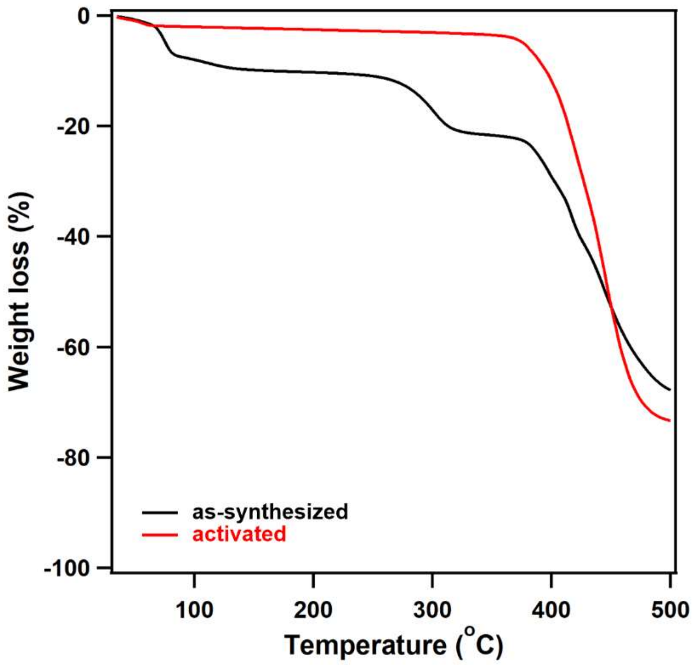

Supplementary Figure 9. TG curves of the as-synthesized and activated **FDC–3**. In the case of the as-synthesized **FDC–3**, the weight loss in the ranges of <100 °C and 250~320 °C corresponded to the loss of methanol and DMA, respectively. **FDC–3a** was thermally stable until 391 °C.

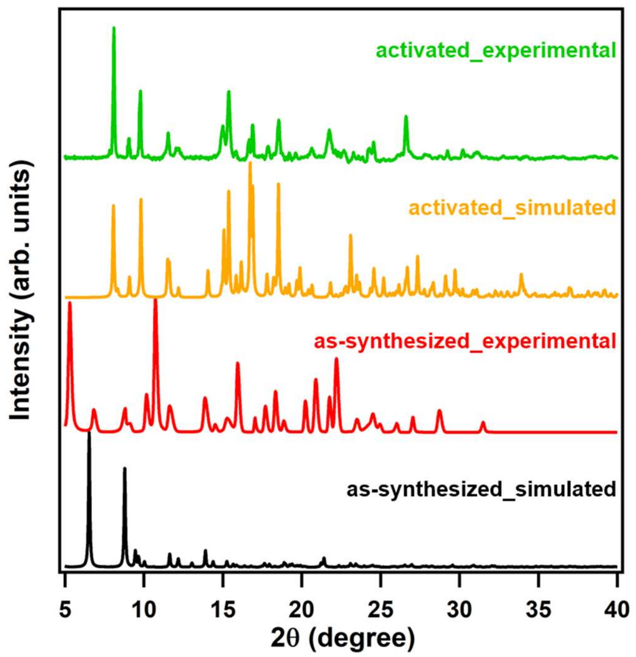

Supplementary Figure 10. PXRD patterns of **FDC–3** (simulated and experimental) and **FDC–3a** (simulated and experimental). The PXRD pattern of as-synthesized **FDC–3** was different from the simulated one because of the structural change upon the loss of volatile solvents (water and methanol) in the crystal-collection process.

Section 4: Gas-sorption behaviors of FDC–3a

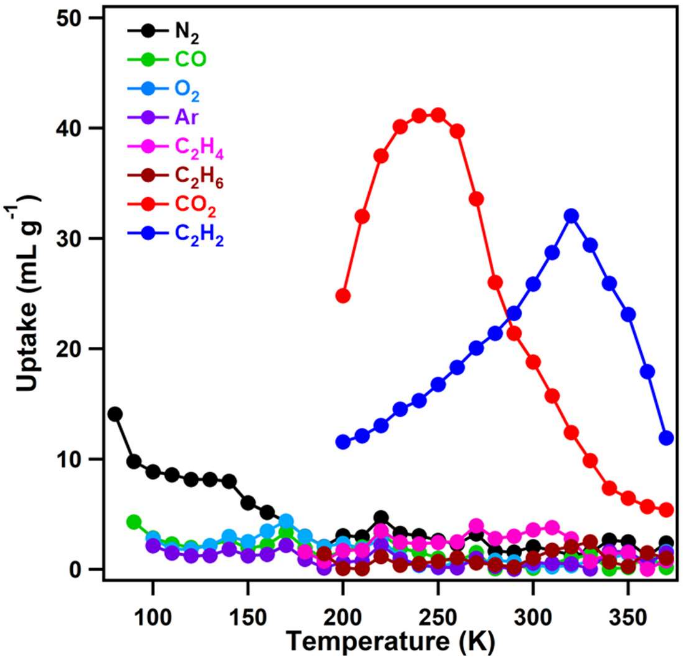

Supplementary Figure 11. Gas-adsorption isobar curves of N_2, CO, CO_2, C_2H_2, O_2, Ar, C_2H_4, and C_2H_6. The isobar measurements were conducted from low to high temperatures. Note that the starting temperatures were ca. 10 K higher than the T_{bp} of the gases. This parameter setting prevented the condensation of the gases on the surface of **FDC–3a**. **FDC–3a** only adsorbed CO_2 and C_2H_2 among the investigated gases.

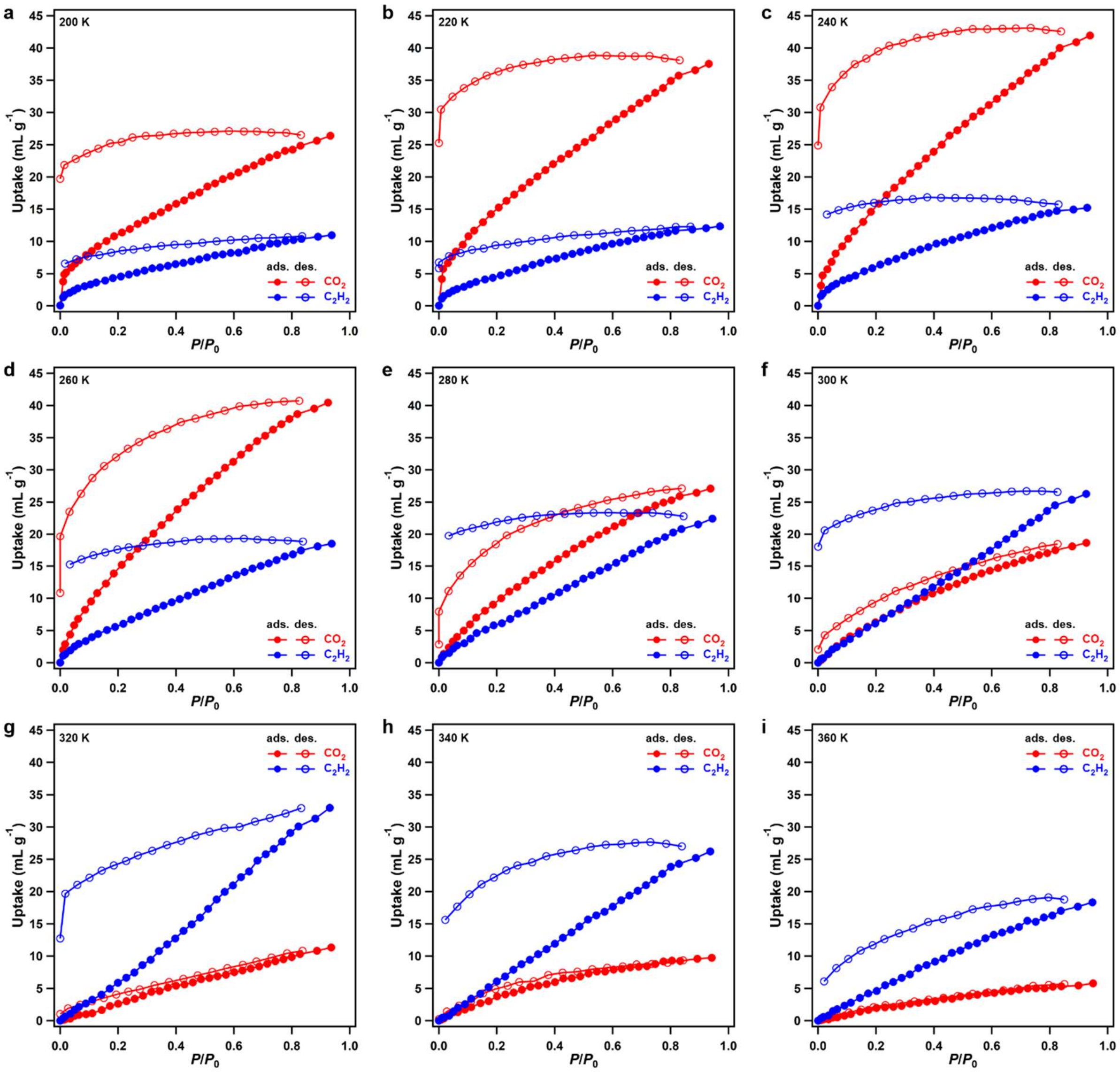

Supplementary Figure 12. CO_2- and C_2H_2-sorption isotherm curves at various temperatures: (a) 200 K; (b) 220 K; (c) 240 K; (d) 260 K; (e) 280 K; (f) 300 K; (g) 320 K; (h) 340 K; (i) 360 K.

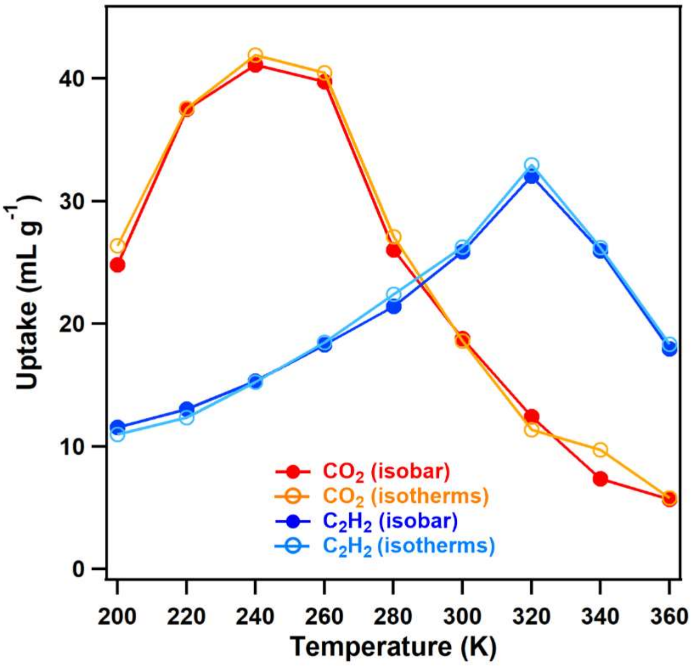

Supplementary Figure 13. The relationship between temperature and maximum CO_2 or C_2H_2 adsorption amounts measured by isobars and isotherms (at $P/P_0 = 1.0$).

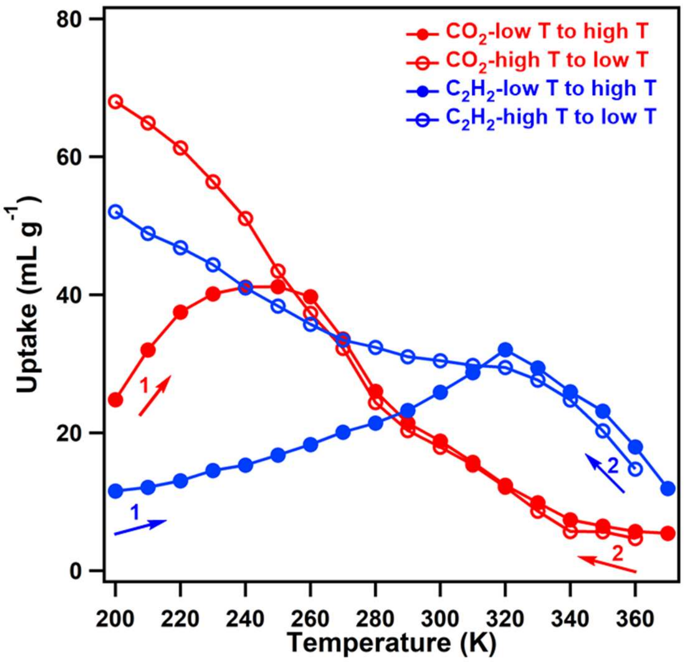

Supplementary Figure 14. CO_2 and C_2H_2 adsorption isobars at temperature directions of 200 K → 370 K → 200 K. The adsorption amounts continuously increased to 68 and 52 mL g^{-1} for CO_2 and C_2H_2, respectively, as the temperature decreased to 200 K. These results suggested that the initial condition of the adsorption is the key to determining the adsorption behaviors. At low temperatures, the diffusion was initially impeded and gradually boosted as increasing the temperature, leading to temperature-assisted adsorption. On the contrary, the diffusion barrier is expected to be lower if the framework was at high temperatures. After adsorbing gases, the diffusion barrier was further decreased. As a result, **FDC–3a** exhibited ordinary adsorption behavior in the temperature range of 370 K → 200 K. These results clearly show a self-promoted adsorption process, in which the pre-adsorbed gas molecules facilitate subsequent adsorption.

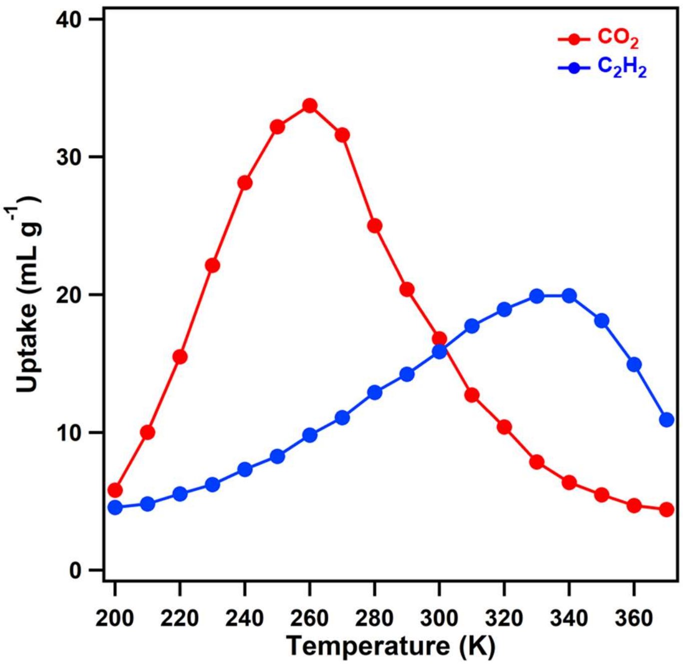

Supplementary Figure 15. Kinetic adsorptions of CO_2 and C_2H_2 at different temperatures. The relative pressure is set to be $P/P_0 = 1.0$ and the exposure time of every temperature is fixed to be 100 s.

Section 5: IAST selectivities of FDC–3a

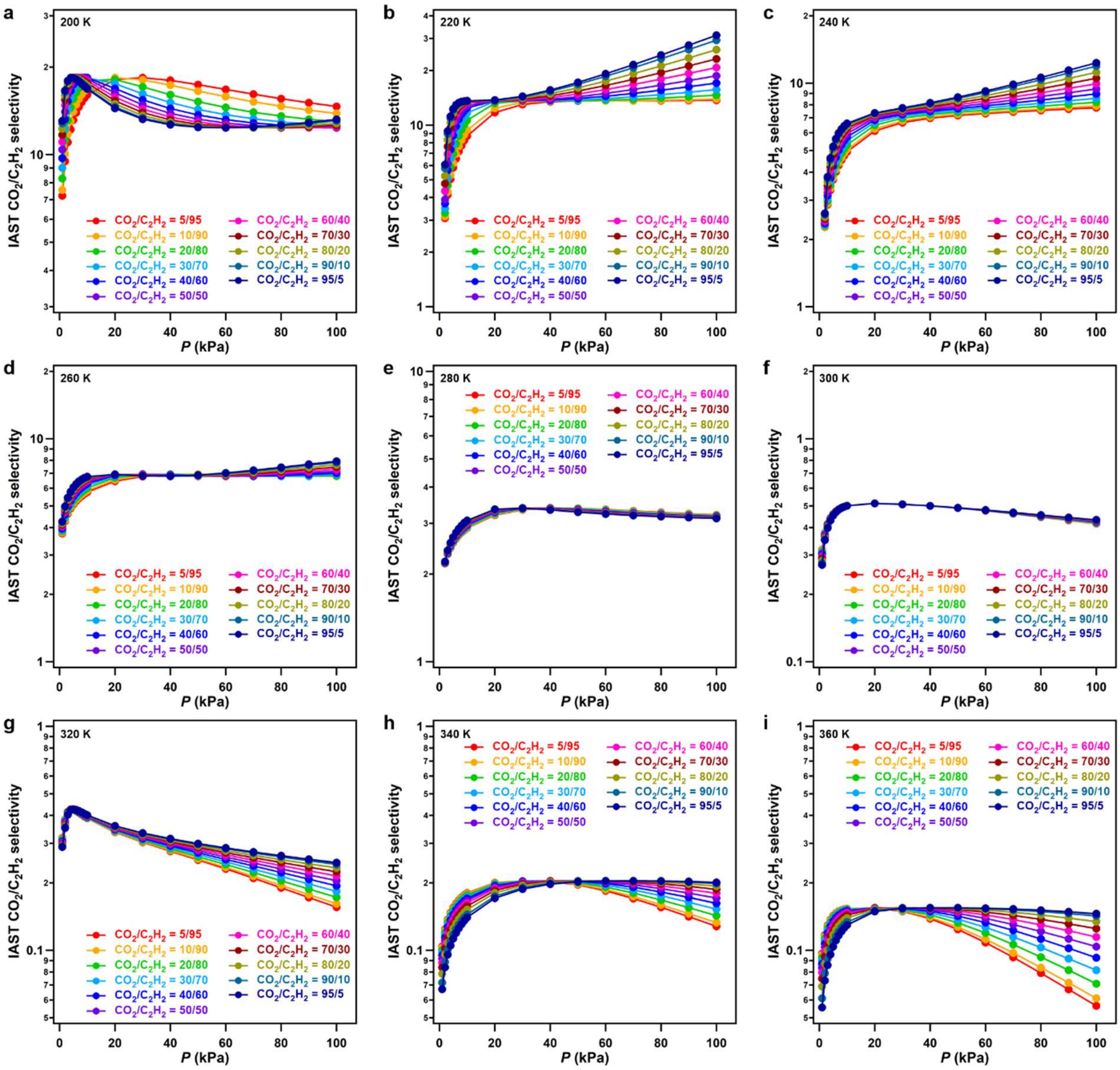

Supplementary Figure 16. IAST CO_2/C_2H_2 selectivity of **FDC–3a** in different CO_2/C_2H_2 ratios at various temperatures: (a) 200 K; (b) 220 K; (c) 240 K; (d) 260 K; (e) 280 K; (f) 300 K; (g) 320 K; (h) 340 K; (i) 360 K.

Section 6: Global pressure–diffusion-rate–adsorption amount curves for FDC–3a

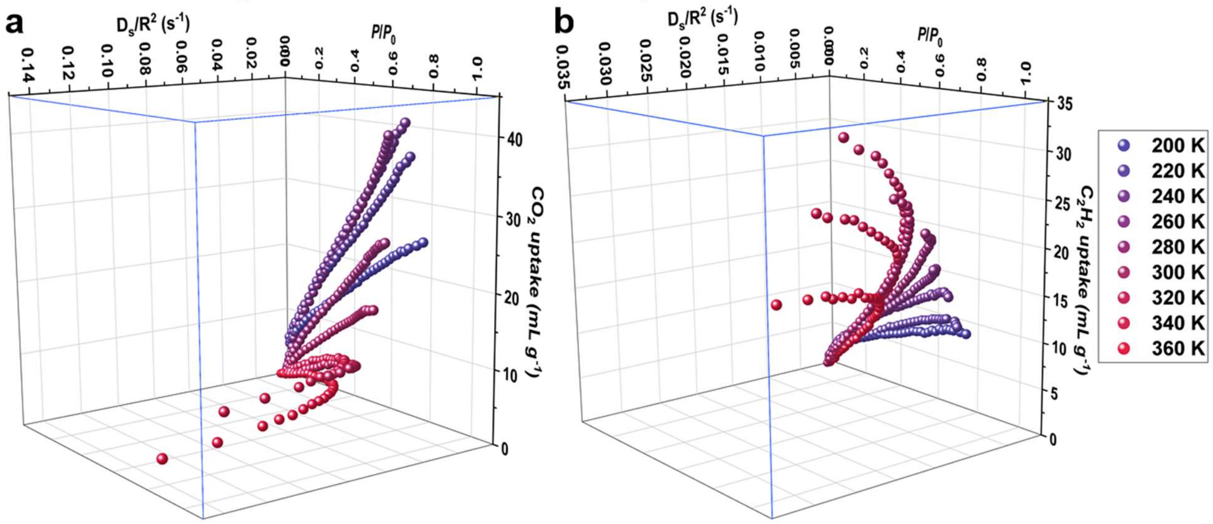

Supplementary Figure 17. (a) Global pressure–diffusion-rate–adsorption-amount (P–D_s/R^2–V) landscape for **FDC–3a** adsorbing CO_2, where R denotes the radius of a PCP particle. (b) P–D_s/R^2–V landscape for **FDC–3a** adsorbing C_2H_2.

Section 7: PXRD and flip-flop mechanism

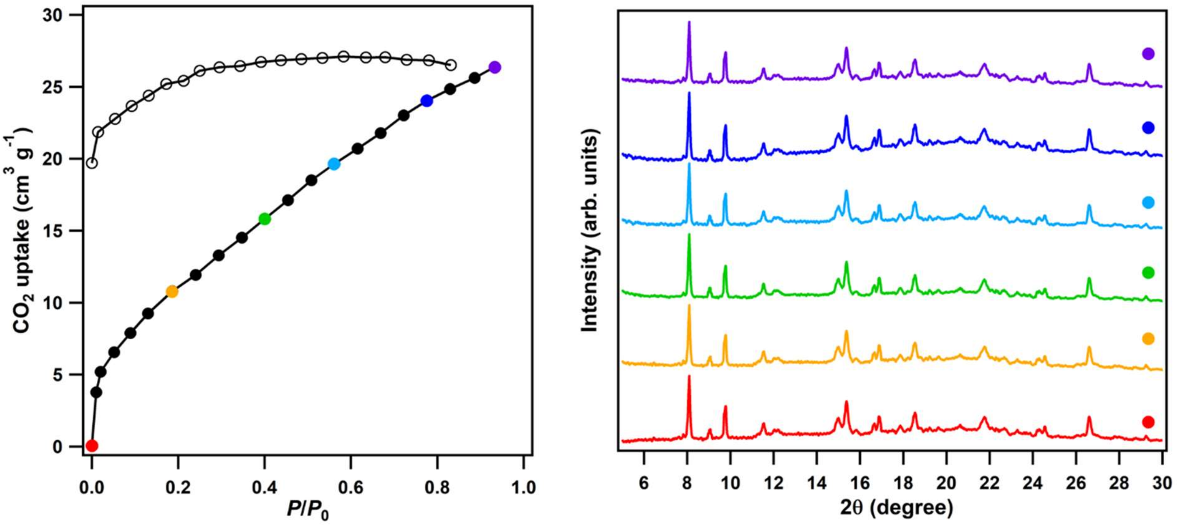

Supplementary Figure 18. Coincident *in-situ* adsorption/PXRD patterns of **FDC–3a** during CO_2 adsorption measured at 200 K at given equilibrium pressures.

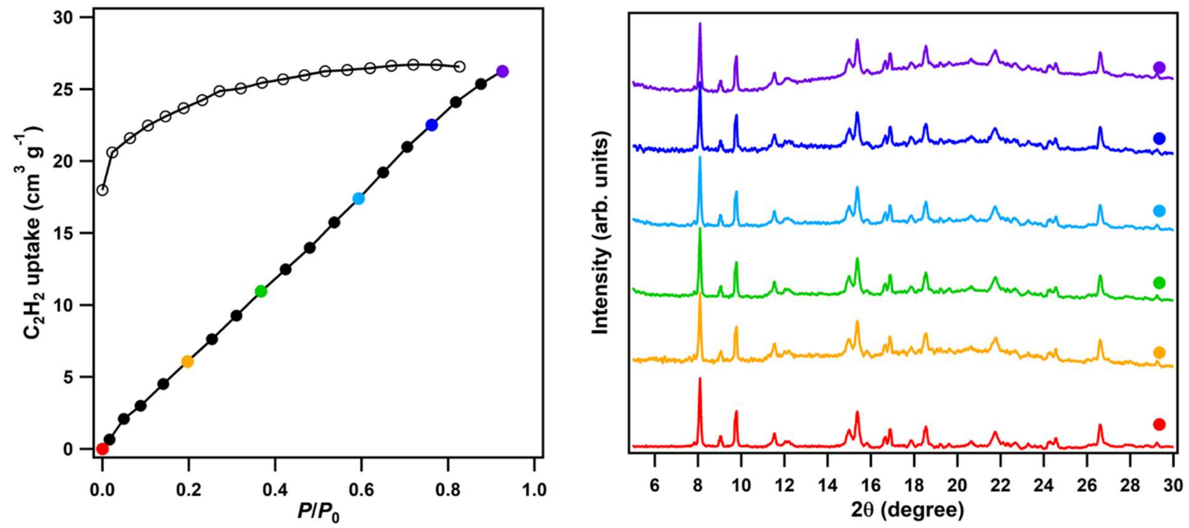

Supplementary Figure 19. Coincident *in-situ* adsorption/PXRD patterns of **FDC–3a** during C_2H_2 adsorption measured at 300 K at given equilibrium pressures.

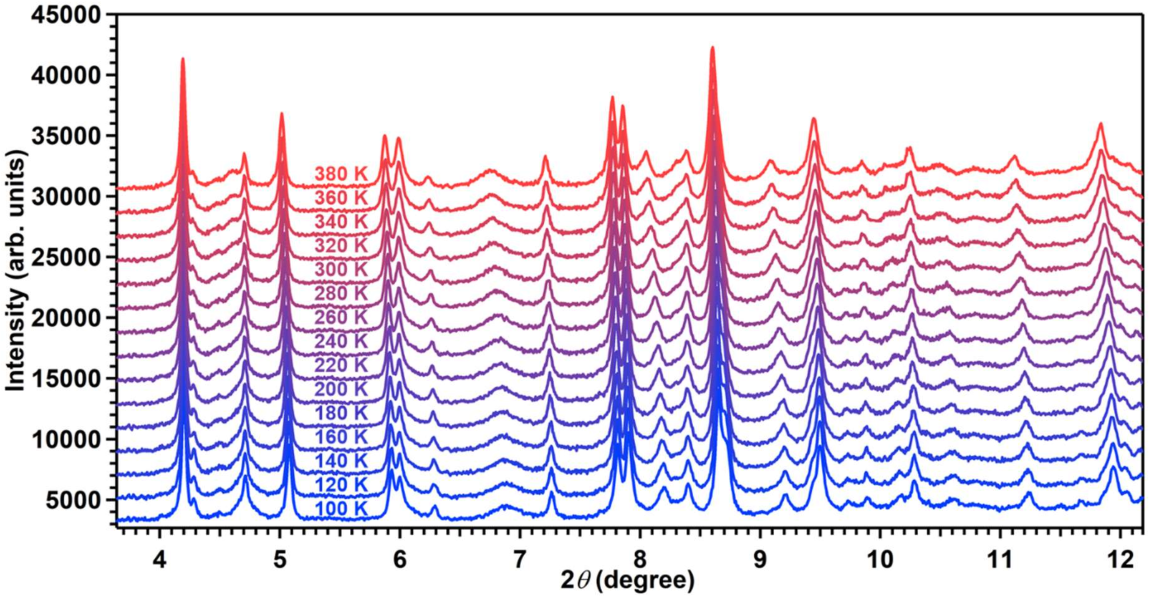

Supplementary Figure 20. Variable-temperature synchrotron PXRD of **FDC–3a** under vacuum conditions in the temperature range from 100 to 380 K.

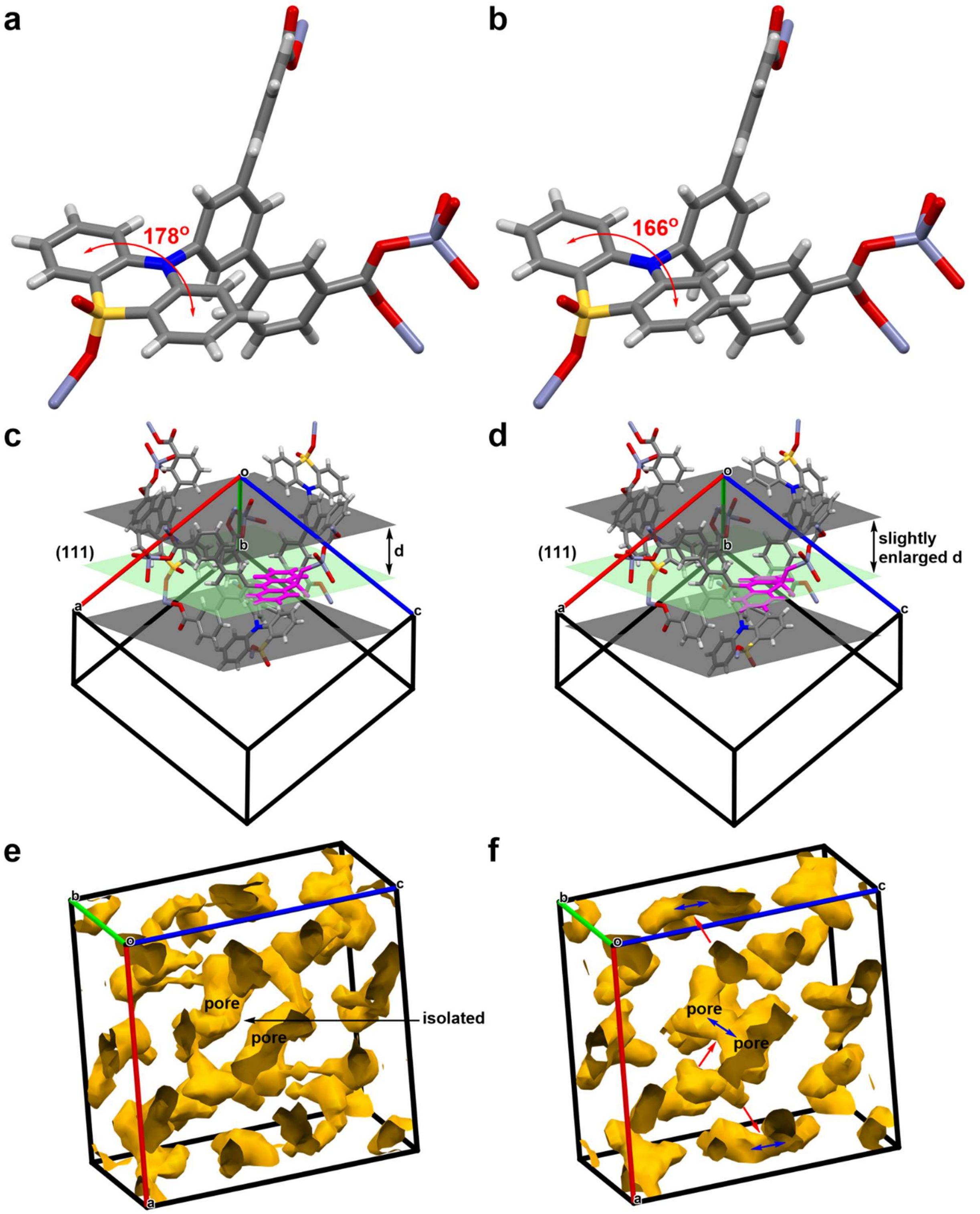

Supplementary Figure 21. Schematic diagram of the effects of the OPTz flipping on the crystal structure. Configuration of OPTz-t3da with different $phenyl_{(OPTz)}$–$phenyl_{(OPTz)}$ dihedral angles of (a) 178º (termed as "static") and (b) 166º (termed as "flipping"), respectively. Structure related to (111) facet in the unit cell of **FDC–3a** under (c) "static" and (d) "flipping" status, respectively.

The OPTz ring in pink color showed the OPTz moiety on the (111) facet (orange plane). The change of pore aperture of **FDC–3a** under (e) "static" and (f) "flipping" status, respectively. The red and blue arrows indicate the pore apertures for diffusion and the diffusion pathways among the pores, respectively. To clearly show the effects of the OPTz flipping on the crystal structure, we manually changed the $phenyl_{(OPTz)}$–$phenyl_{(OPTz)}$ dihedral angles on the OPTz ring from 178° to 166°, while keeping other parts of the crystal structure the same as the initial structure. In these two structures, the structure of the framework kept the same, whereas the OPTz moieties showed different $phenyl_{(OPTz)}$–$phenyl_{(OPTz)}$ dihedral angles. Thus, the two structures could stand for the non-flipping (static) and flipping modes of **FDC–3a** at low and high temperatures, respectively. The "flipping" structure revealed a slight expansion of the [111] axis compared to the "static" structure, in good agreement with the VT-PXRD results that the peaks corresponding to the (111) facet shifted to a lower angle. Since one OPTz moiety is on the (111) plane, this tiny expansion of [111] distances could be correlated with the extent of thermal flipping of the OPTz moiety. Notably, the "flipping" structure showed a remarkable enlargement of pore aperture for the diffusion of CO_2 and C_2H_2 compared to the "static" structure, which indicated that the "flipping" structure allowed accelerating the diffusion of CO_2 and C_2H_2 in response to increasing temperature.

Section 8: Solid-state NMR

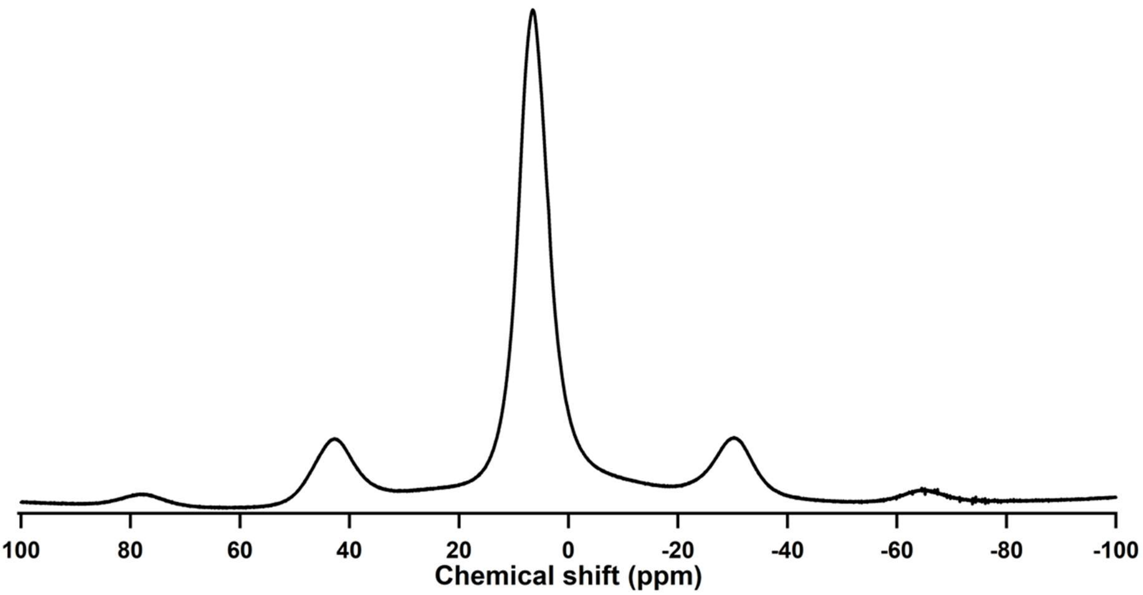

Supplementary Figure 22. ^{1}H MAS spectrum of **FDC–3a**.

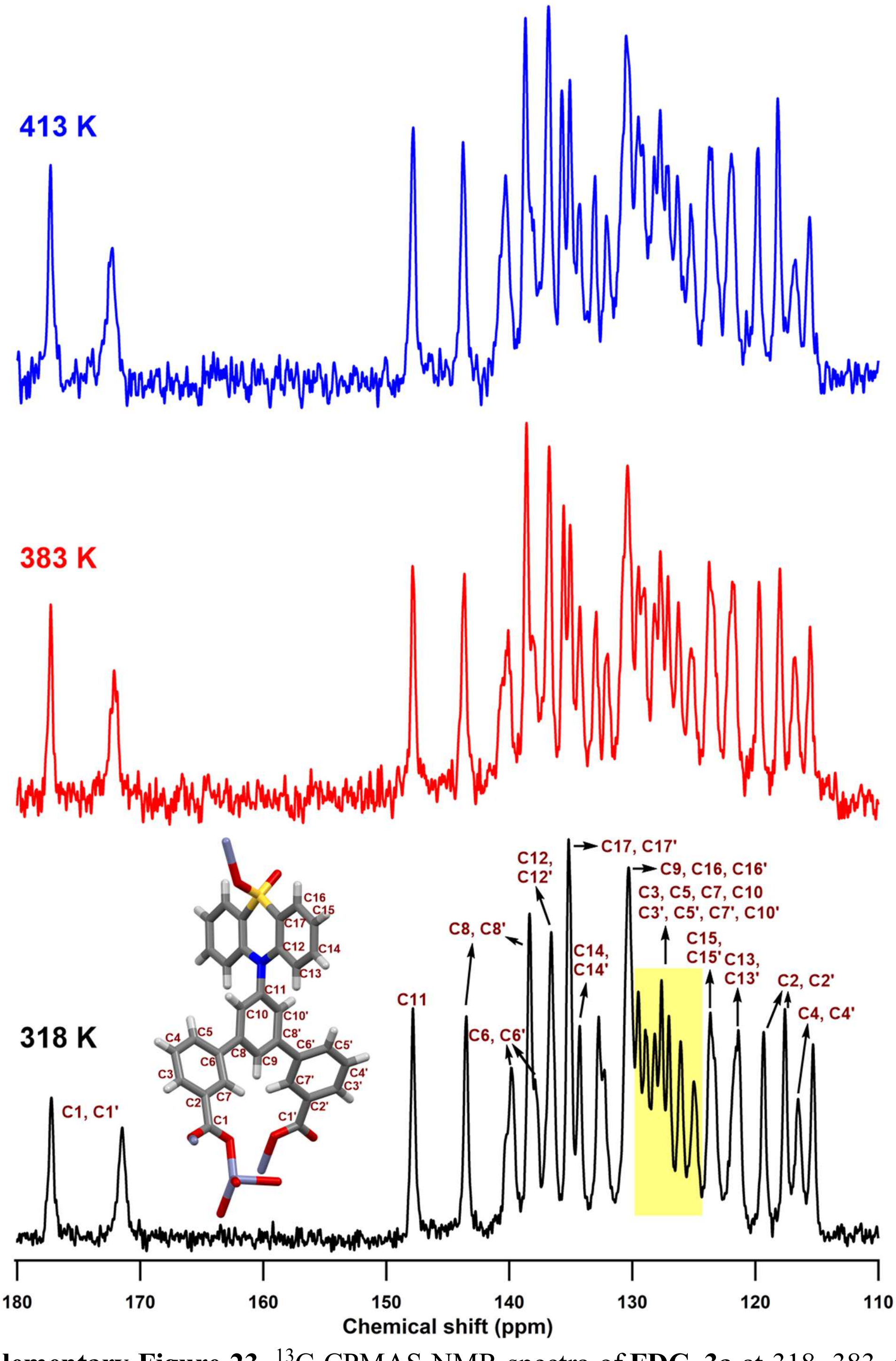

Supplementary Figure 23. ^{13}C CPMAS NMR spectra of **FDC–3a** at 318, 383, and 413 K. The peaks at high-temperature conditions remained clear, indicating the high thermal stability of **FDC–3a**.

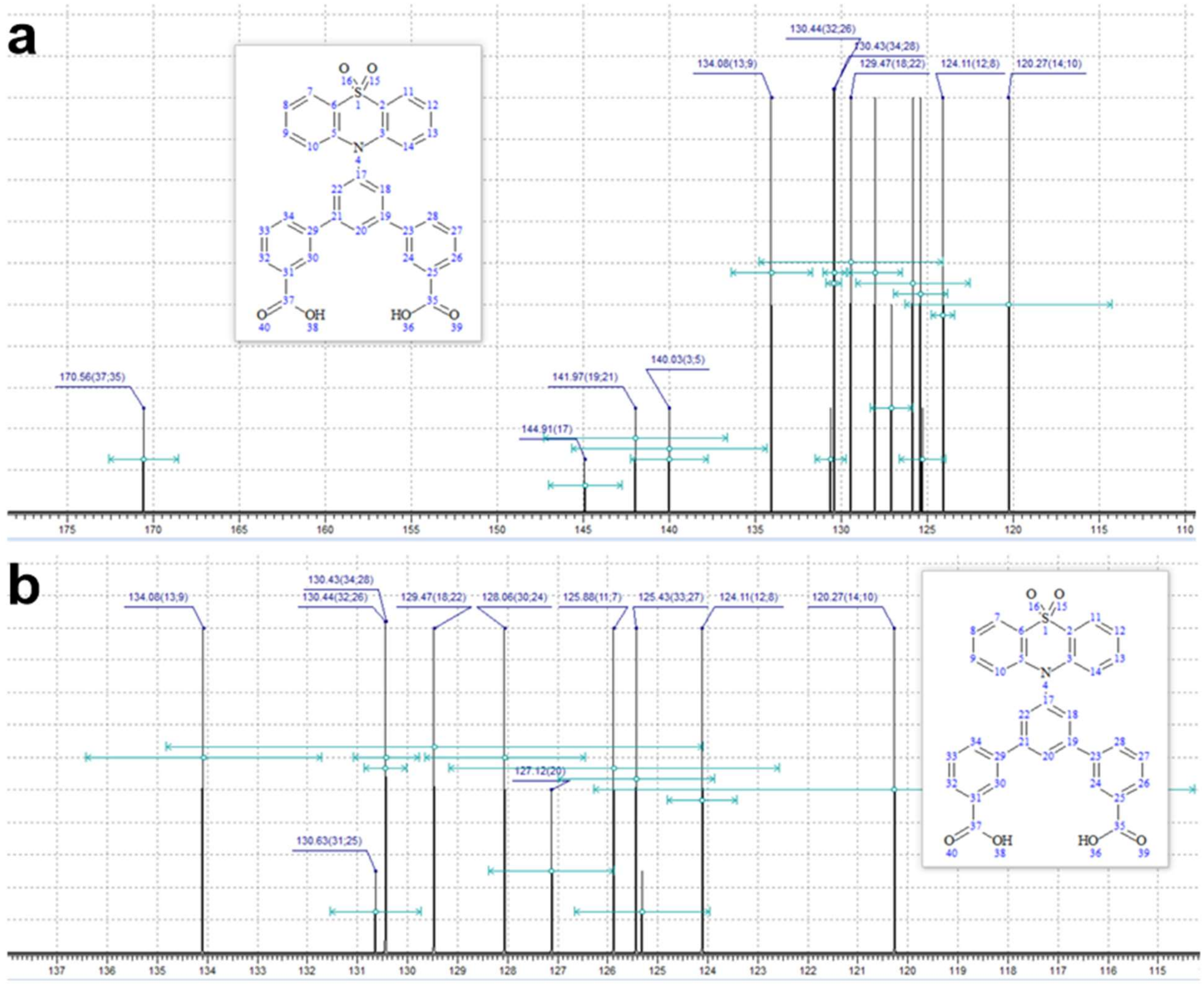

Supplementary Figure 24. Calculated solution ^{13}C NMR (a) full spectrum and (b) enlarged spectrum of the OPTz-t3da ligand using ACD NMR Workbook Suite.

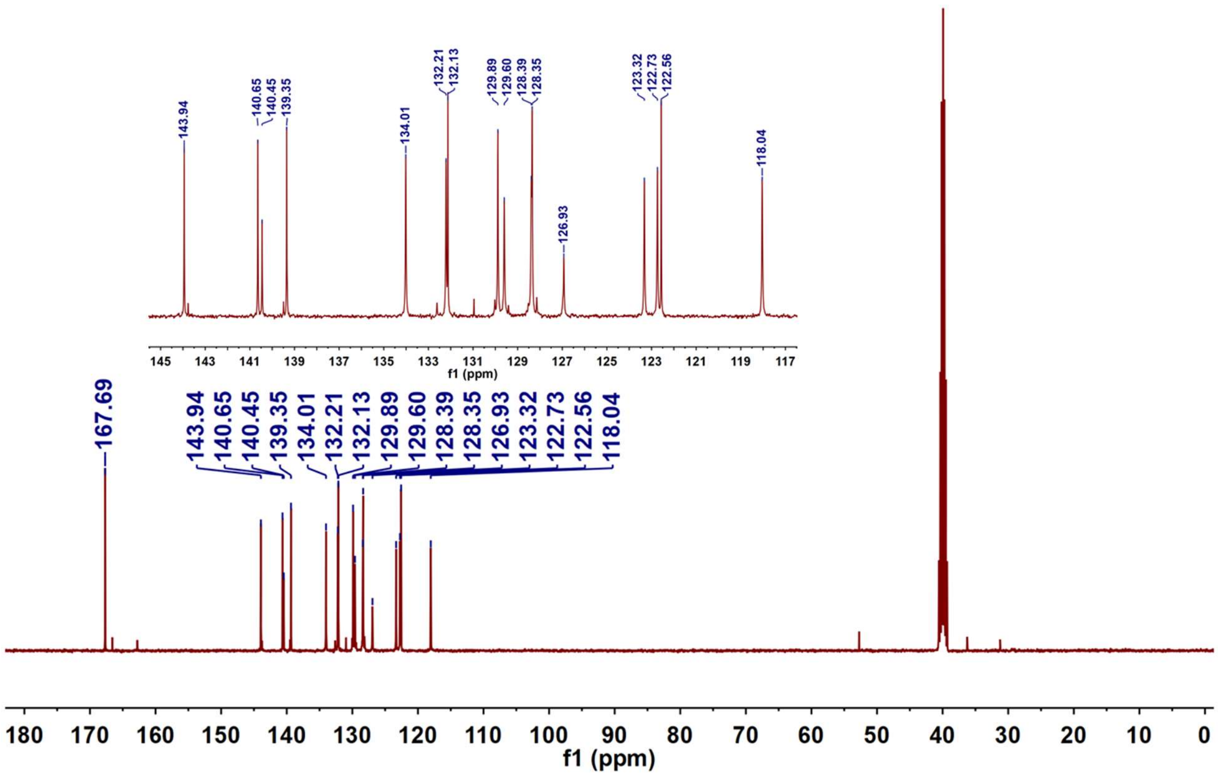

Supplementary Figure 25. Solution ^{13}C CPMAS NMR spectrum of OPTz-t3da ligand.

Section 9: Theoretical calculation of the diffusion energies and rates

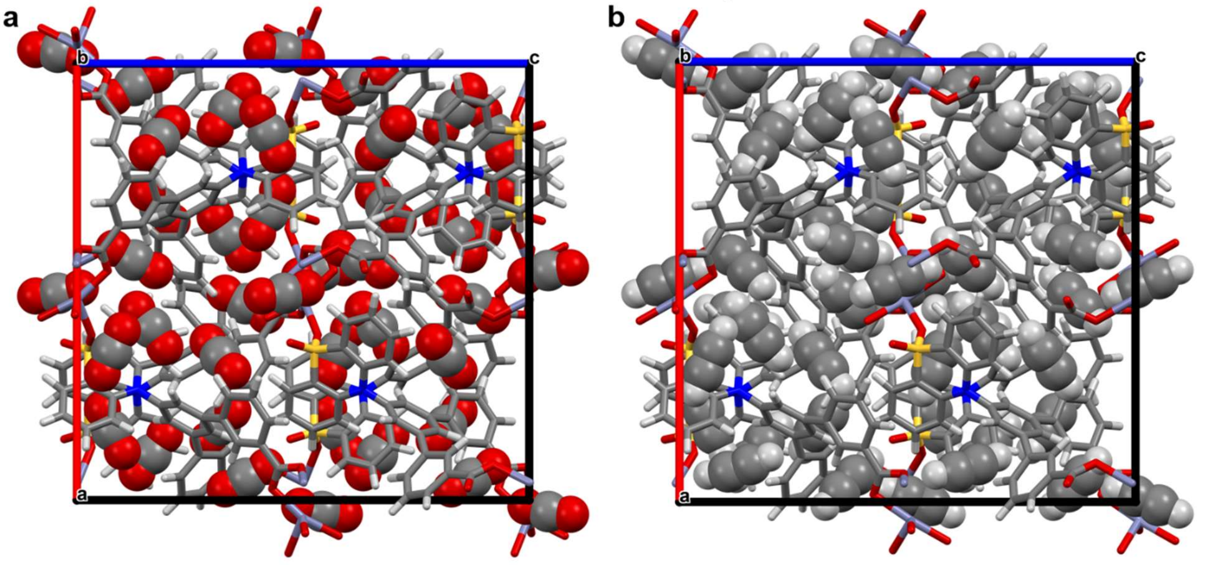

Supplementary Figure 26. Simulated structures of (a) CO_2-loading saturated and (b) C_2H_2-loading saturated **FDC–3a**.

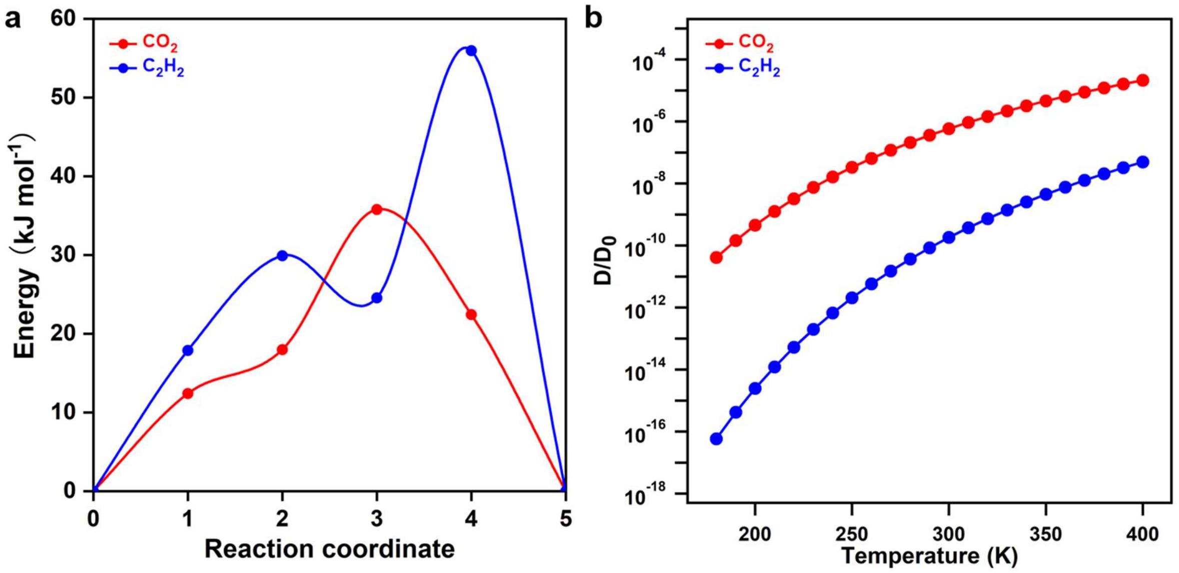

Supplementary Figure 27. (a) Reaction coordinate–energy plot to determine the diffusion energies for the CO_2- and C_2H_2-adsorbed phases of **FDC–3a** (35.8 and 55.9 kJ mol^{-1}, respectively). (b) Calculated diffusion rates of **FDC–3a** under CO_2- and C_2H_2-adsorbed phases. The diffusion rates are calculated by the Arrhenius equation $D/D_0 = \exp(-E_a/RT)$, where E_a is the diffusion barrier.

Section 10: Gas separation experiments for FDC–3a

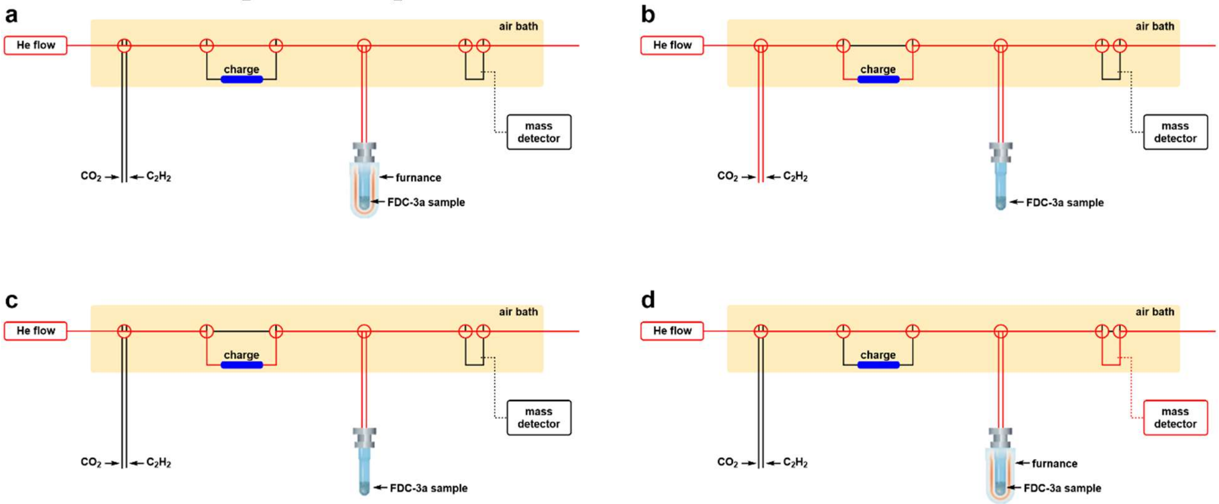

Supplementary Figure 28. Flow path diagram of TPD gas separation experiment for the process (a) No. 1, (b) No. 2, (c) No. 3 (d) No. 4.

Note: Experimental procedure for gas separation with flow diagrams:

No. 1. Activating the sample in the cell under He flow at 393 K for 2 h (a).

No. 2. Mixed gas flowing at 240 or 320 K (b).

No. 3. Flowing away remaining gases in the gas line and sample tube with He flush (c).

No. 4. Heating to 393 K to desorb gas from the sample and mass test (d).

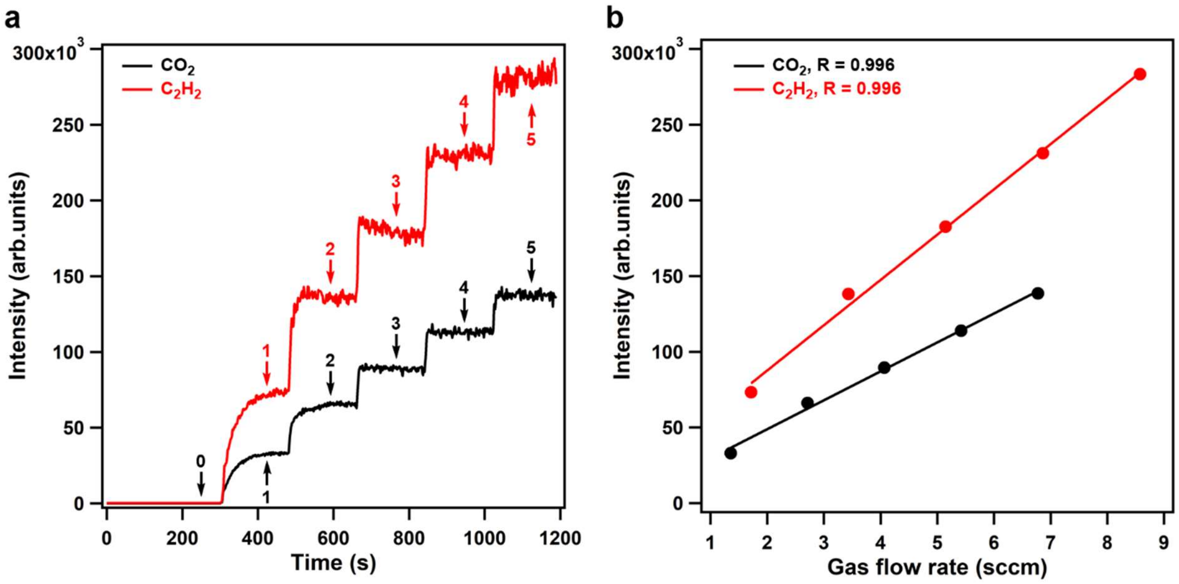

Supplementary Figure 29. Calibration of the TPD signals in the mass detector. (a) Raw data of the mass detector for five times of gas dosing. (b) Correlation of the electric intensity in the mass detector with the gas flow rate of CO_2/C_2H_2 in the feed gas.

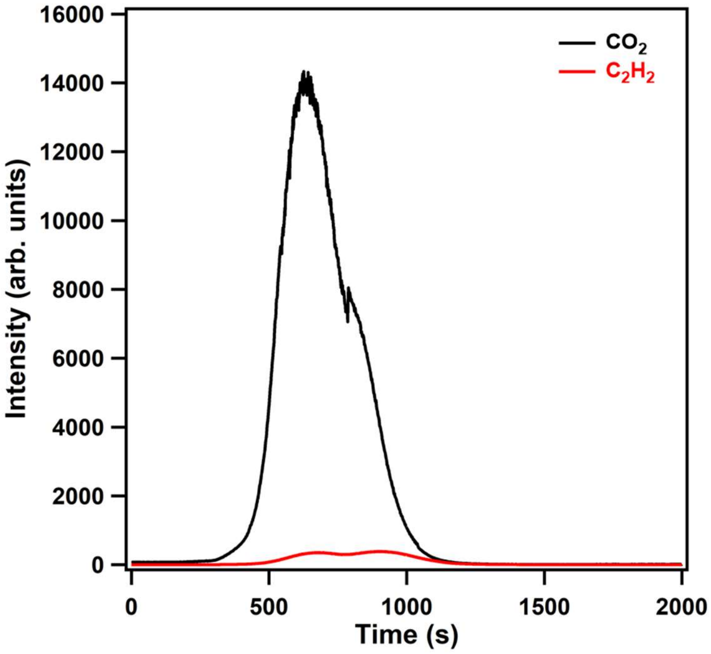

Supplementary Figure 30. TPD spectra of adsorbed gas in **FDC–3a** at 1 h exposure time at 240 K. The feed-gas ratio was CO_2:C_2H_2 = 93.8:6.2.

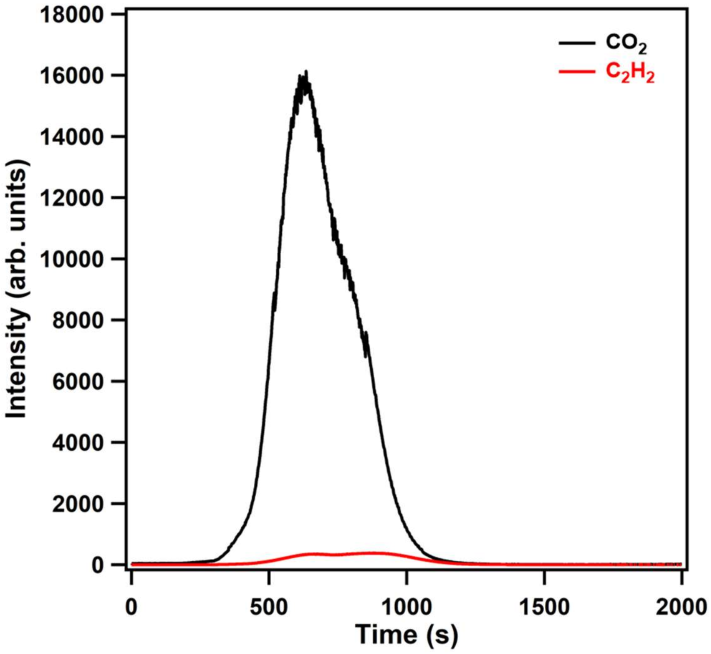

Supplementary Figure 31. TPD spectra of adsorbed gas in **FDC–3a** at 1 h exposure time at 240 K. The feed-gas ratio was CO_2:C_2H_2 = 87.7:12.3.

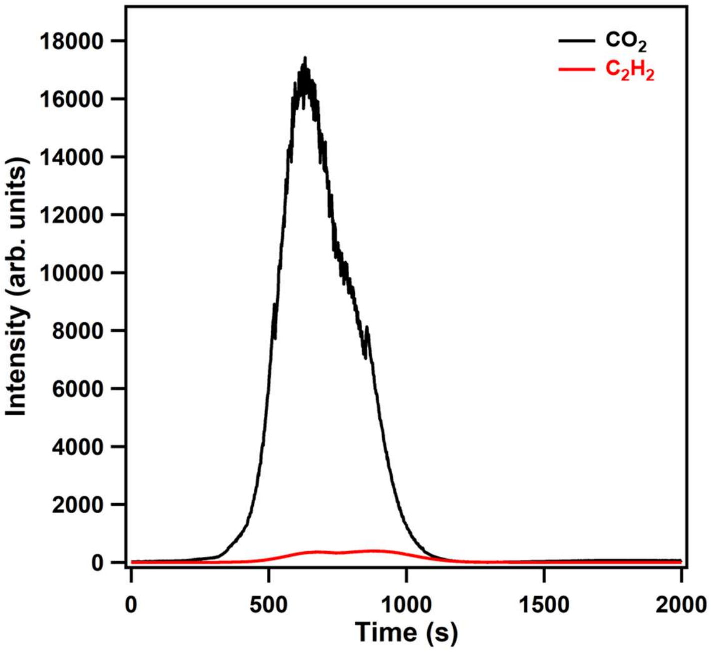

Supplementary Figure 32. TPD spectra of adsorbed gas in **FDC–3a** at 1 h exposure time at 240 K. The feed-gas ratio was CO_2:C_2H_2 = 76.0:24.0.

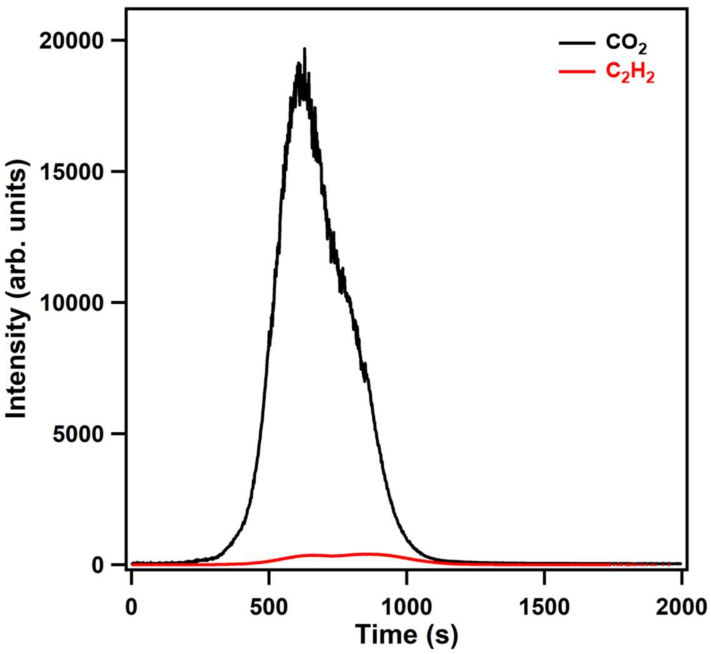

Supplementary Figure 33. TPD spectra of adsorbed gas in **FDC–3a** at 1 h exposure time at 240 K. The feed-gas ratio was CO_2:C_2H_2 = 64.8:35.2.

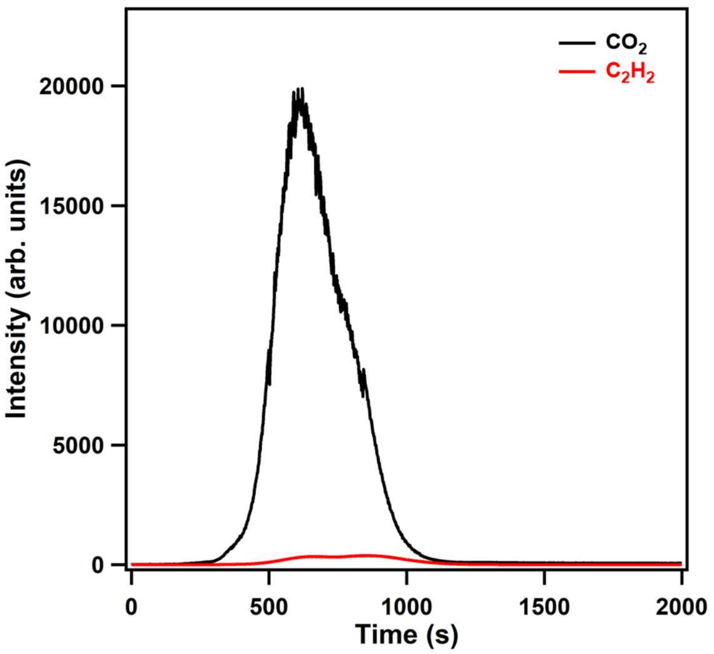

Supplementary Figure 34. TPD spectra of adsorbed gas in **FDC–3a** at 1 h exposure time at 240 K. The feed-gas ratio was CO_2:C_2H_2 = 54.2:45.8.

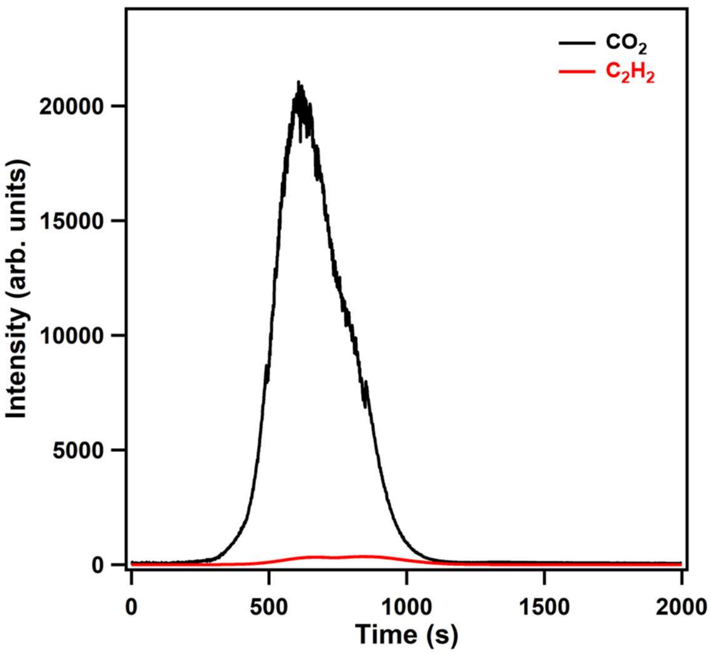

Supplementary Figure 35. TPD spectra of adsorbed gas in **FDC–3a** at 1 h exposure time at 240 K. The feed-gas ratio was CO_2:C_2H_2 = 44.1:55.9.

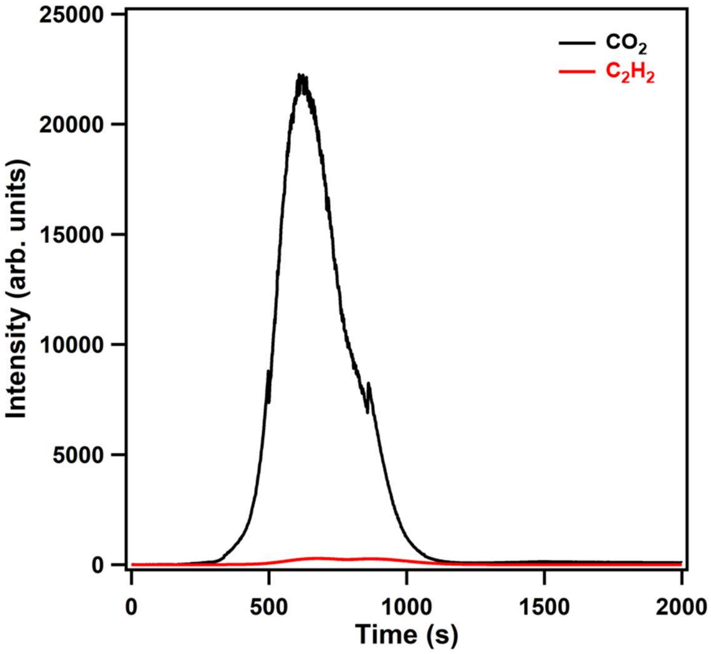

Supplementary Figure 36. TPD spectra of adsorbed gas in **FDC–3a** at 1 h exposure time at 240 K. The feed-gas ratio was CO_2:C_2H_2 = 34.5:65.5.

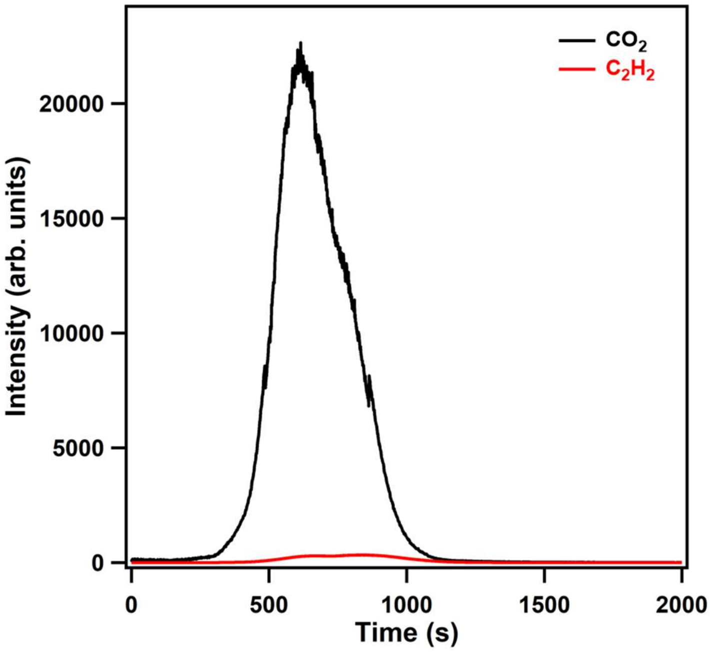

Supplementary Figure 37. TPD spectra of adsorbed gas in **FDC–3a** at 1 h exposure time at 240 K. The feed-gas ratio was CO_2:C_2H_2 = 25.3:74.7.

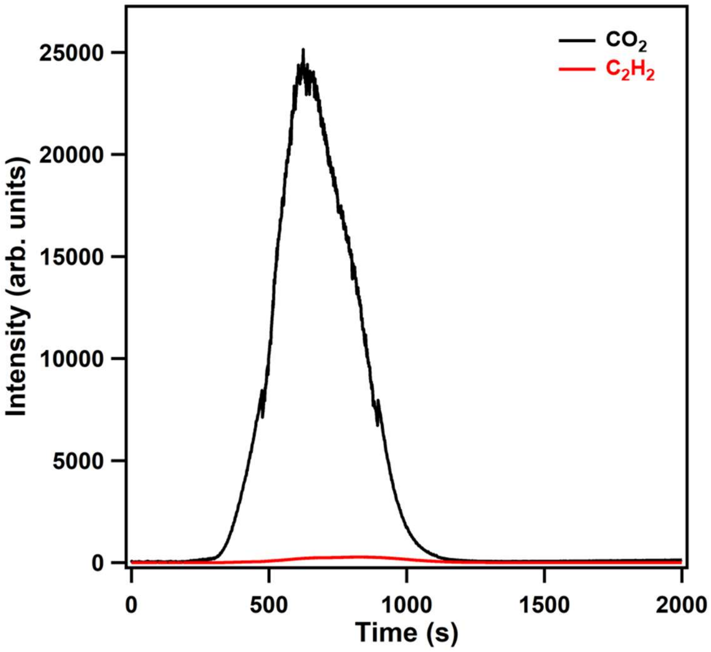

Supplementary Figure 38. TPD spectra of adsorbed gas in **FDC–3a** at 1 h exposure time at 240 K. The feed-gas ratio was CO_2:C_2H_2 = 16.5:83.5.

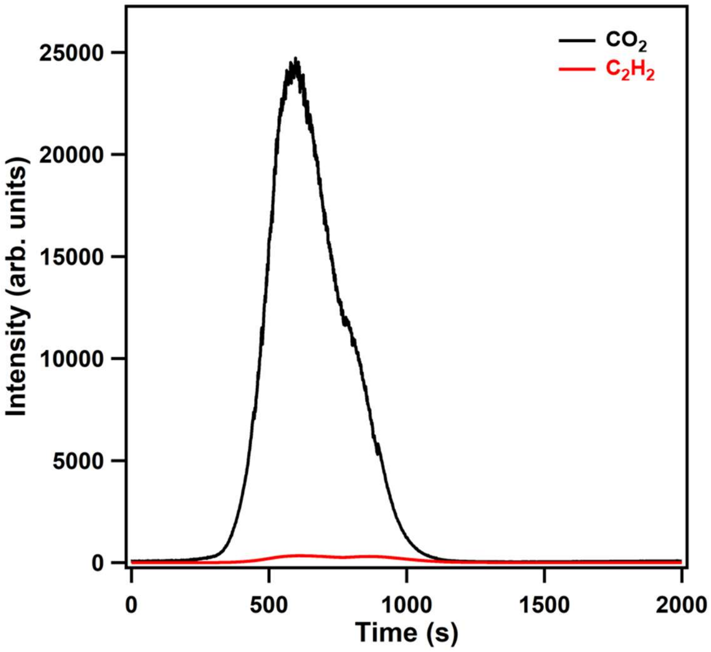

Supplementary Figure 39. TPD spectra of adsorbed gas in **FDC–3a** at 1 h exposure time at 240 K. The feed-gas ratio was CO_2:C_2H_2 = 8.1:91.9.

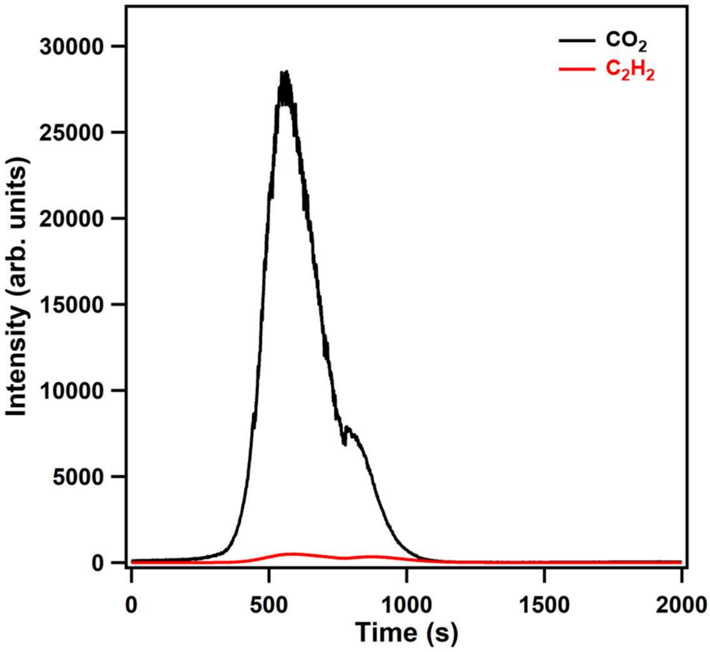

Supplementary Figure 40. TPD spectra of adsorbed gas in **FDC–3a** at 1 h exposure time at 240 K. The feed-gas ratio was CO_2:C_2H_2 = 4.0:96.0.

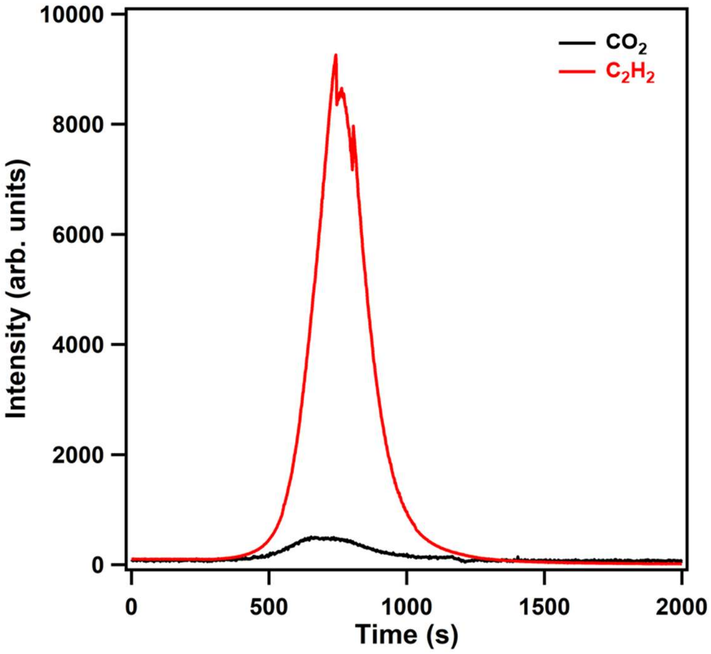

Supplementary Figure 41. TPD spectra of adsorbed gas in **FDC–3a** at 1 h exposure time at 320 K. The feed-gas ratio was CO_2:C_2H_2 = 93.8:6.2.

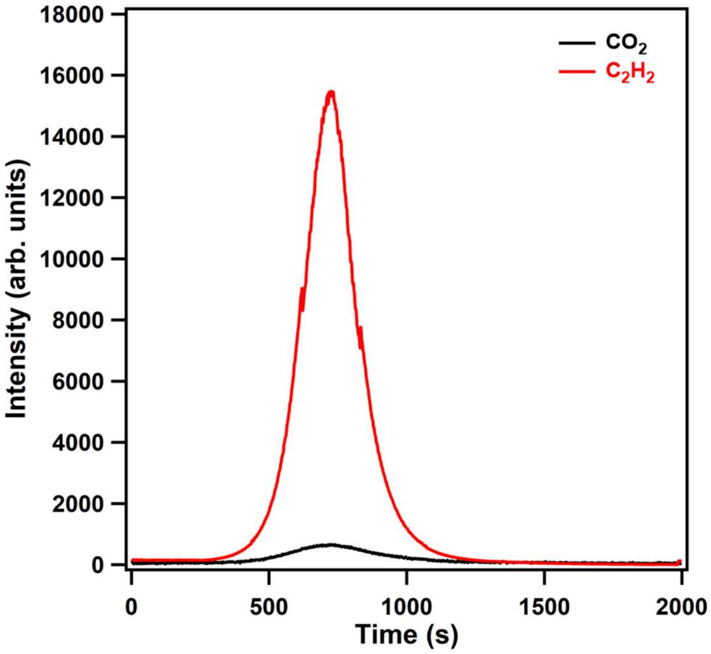

Supplementary Figure 42. TPD spectra of adsorbed gas in **FDC–3a** at 1 h exposure time at 320 K. The feed-gas ratio was CO_2:C_2H_2 = 87.7:12.3.

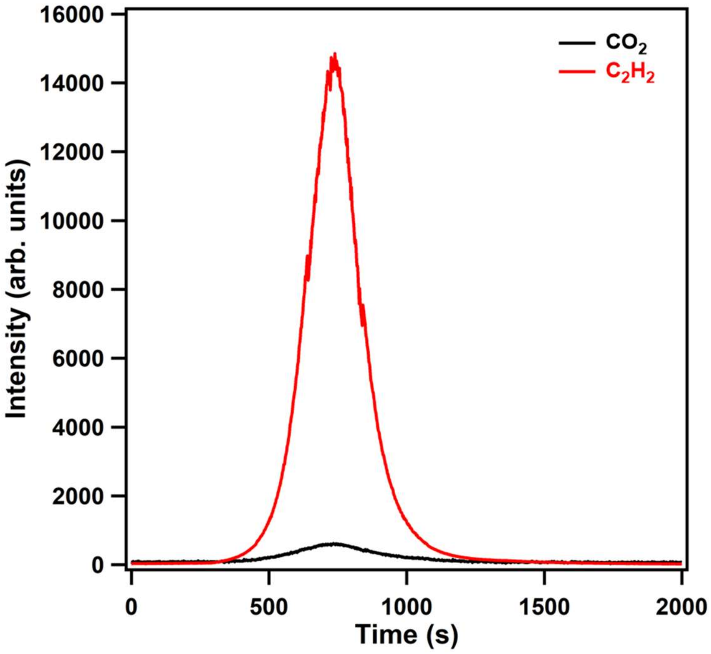

Supplementary Figure 43. TPD spectra of adsorbed gas in **FDC–3a** at 1 h exposure time at 320 K. The feed-gas ratio was CO_2:C_2H_2 = 76.0:24.0.

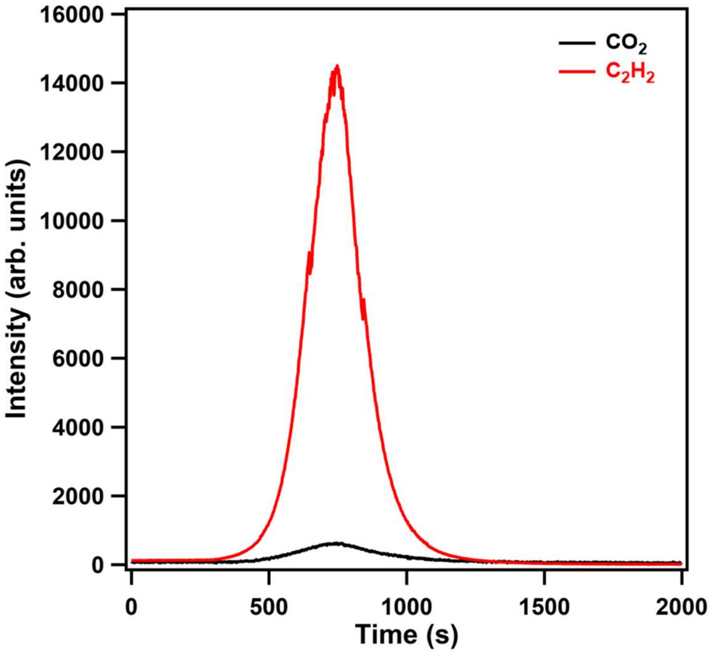

Supplementary Figure 44. TPD spectra of adsorbed gas in **FDC–3a** at 1 h exposure time at 320 K. The feed-gas ratio was CO_2:C_2H_2 = 64.8:35.2.

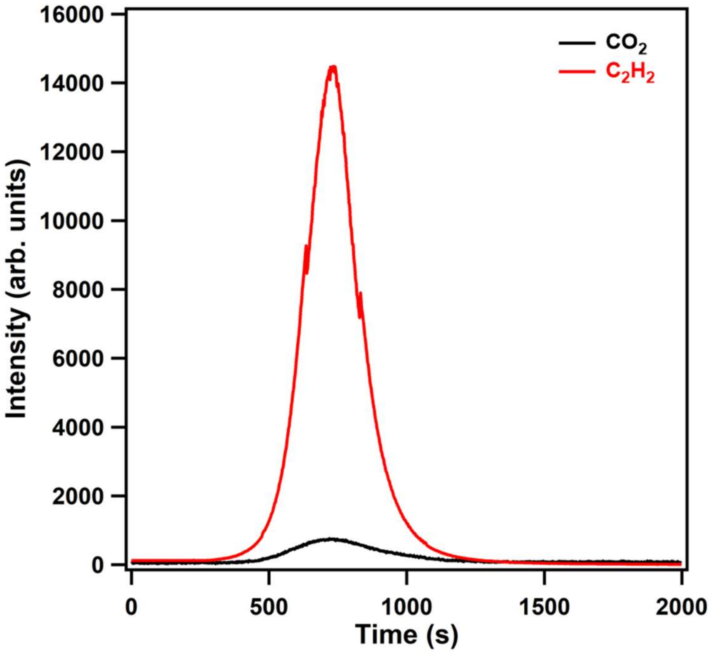

Supplementary Figure 45. TPD spectra of adsorbed gas in **FDC–3a** at 1 h exposure time at 320 K. The feed-gas ratio was CO_2:C_2H_2 = 54.2:45.8.

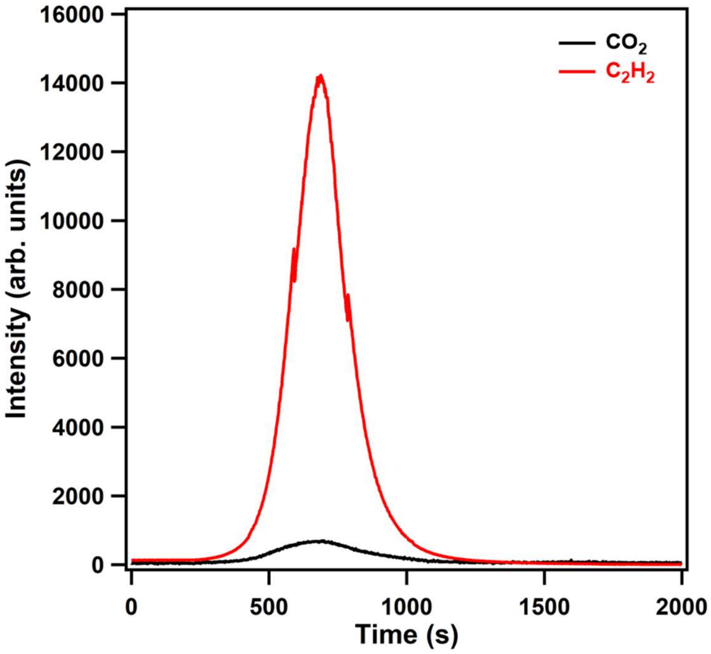

Supplementary Figure 46. TPD spectra of adsorbed gas in **FDC–3a** at 1 h exposure time at 320 K. The feed-gas ratio was CO_2:C_2H_2 = 44.1:55.9.

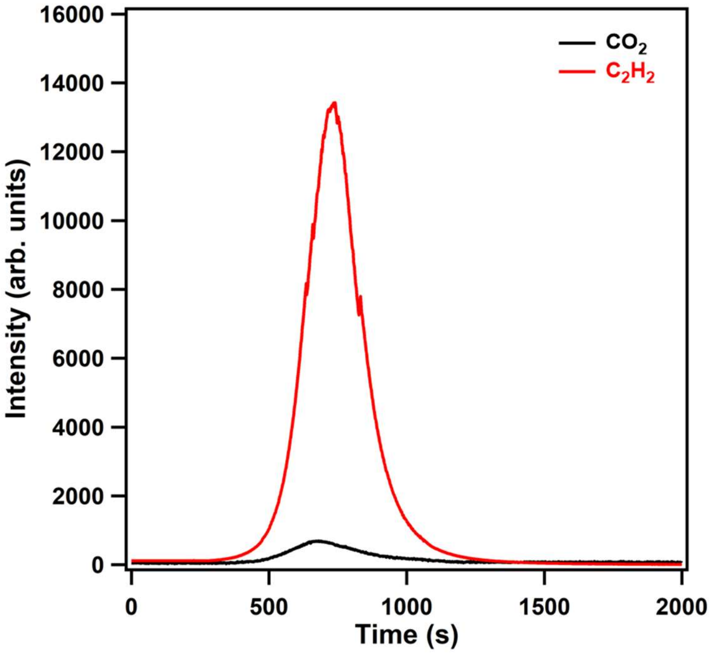

Supplementary Figure 47. TPD spectra of adsorbed gas in **FDC–3a** at 1 h exposure time at 320 K. The feed-gas ratio was CO_2:C_2H_2 = 34.5:65.5.

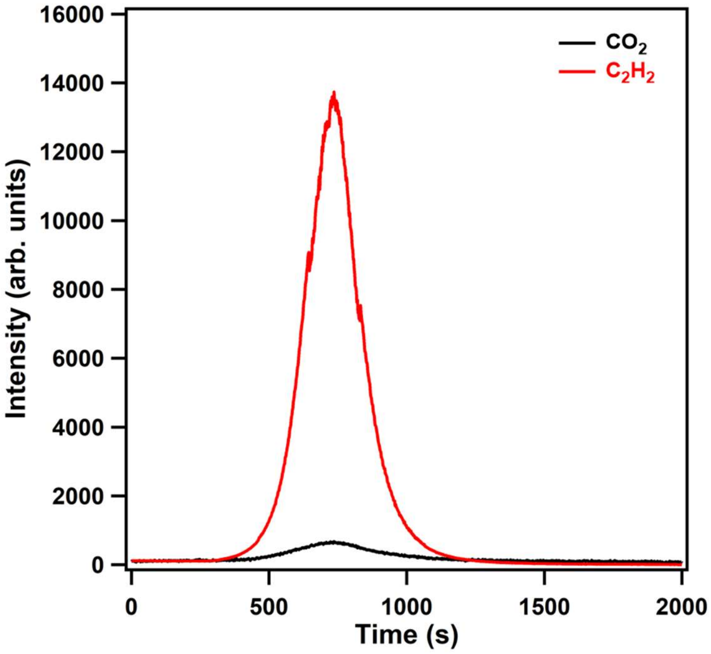

Supplementary Figure 48. TPD spectra of adsorbed gas in **FDC–3a** at 1 h exposure time at 320 K. The feed-gas ratio was CO_2:C_2H_2 = 25.3:74.7.

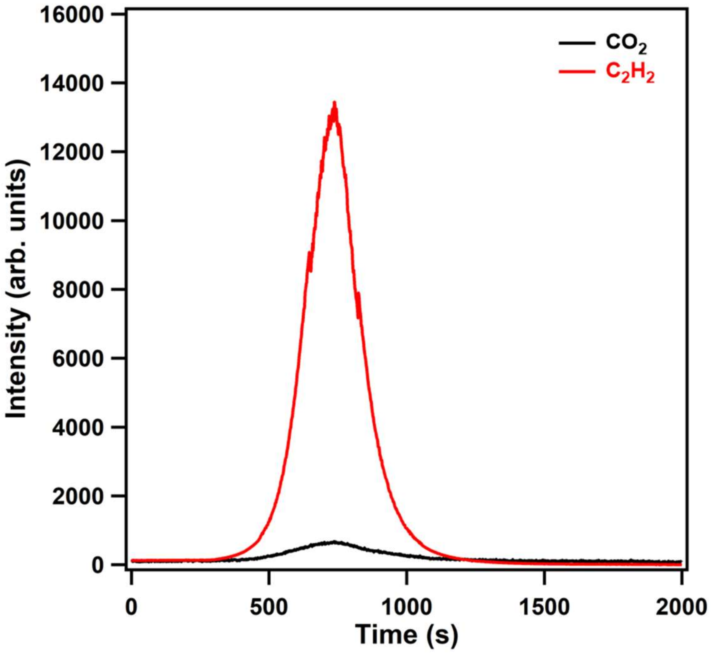

Supplementary Figure 49. TPD spectra of adsorbed gas in **FDC–3a** at 1 h exposure time at 320 K. The feed-gas ratio was CO_2:C_2H_2 = 16.5:83.5.

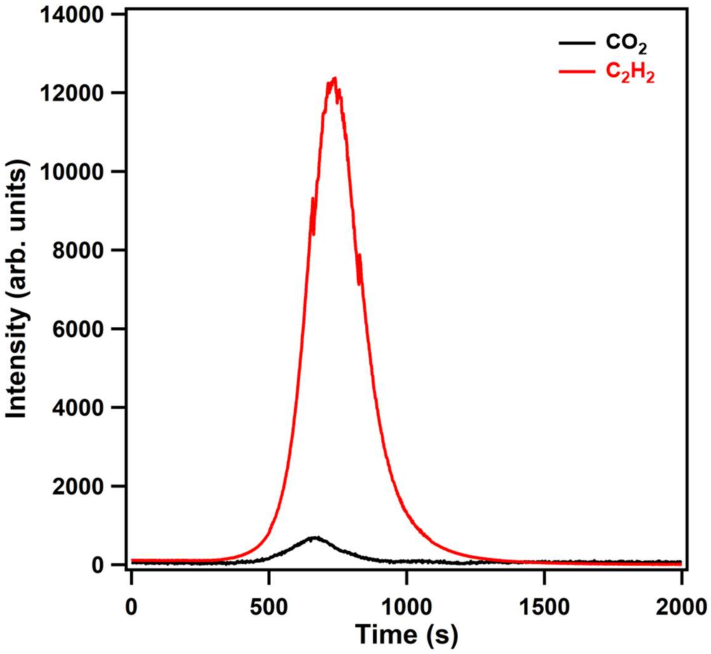

Supplementary Figure 50. TPD spectra of adsorbed gas in **FDC–3a** at 1 h exposure time at 320 K. The feed-gas ratio was CO_2:C_2H_2 = 8.1:91.9.

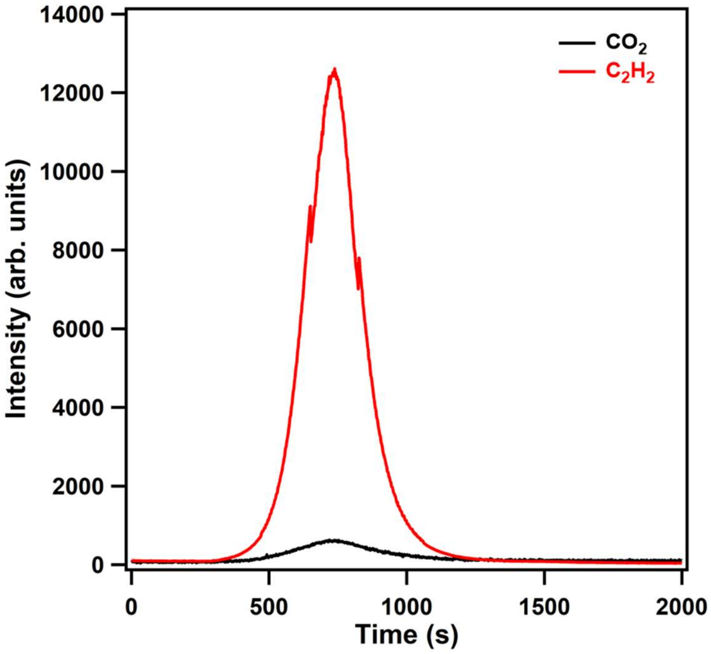

Supplementary Figure 51. TPD spectra of adsorbed gas in **FDC–3a** at 1 h exposure time at 320 K. The feed-gas ratio was CO_2:C_2H_2 = 4.0:96.0.

Supplementary Tables

Section 1: Crystallographic data for as-synthesized and activated FDCs

Supplementary Table 1. Crystallographic data and structural refinement summary for as-synthesized FDC–3.

	as-synthesized **FDC–3**
crystal system	monoclinic
space group	$P2_1/c$ (#14)
empirical formula	$C_{32}H_{21}NO_7SZn$
a (Å)	18.5163(4)
b (Å)	20.0936(3)
c (Å)	11.7529(3)
α (°)	90
β (°)	98.683(2)
γ (°)	90
V (Å^3)	4322.66(17)
Z, d_{calcd} (g cm^{-3})	4, 0.966
diffractometer	CCD
μ (cm^{-1})	1.522
radiation type	Cu $K\alpha$
radiation wavelength (Å)	1.54184
F(000)	1288
goodness of fit	1.095
temperature (K)	293(2)
number of reflections collected/unique	8566/7072
R_{int}	0.0676
R_1 ($I > 2.00\sigma(I)$)[a]	0.0578
wR_2 ($I > 2.00\sigma(I)$)[b]	0.1796
CCDC deposition number	2236266

[a]$R_1 = \Sigma||F_o|-|F_c||/\Sigma|F_o|$, [b]$wR_2 = [\Sigma w|F_o^2-F_c^2|^2/\Sigma w(F_o^2)^2]^{1/2}$

Supplementary Table 2. Crystallographic data and structural refinement summary for activated FDC–3a.

	activated **FDC–3a**
crystal system	orthorhombic
space group	Pbca (#61)
empirical formula	$C_{32}H_{19}NO_6SZn$
a (Å)	21.180(4)
b (Å)	11.180(2)
c (Å)	21.910(4)
α (°)	90
β (°)	90
γ (°)	90
V (Å^3)	5188.1(18)
Z, d_{calcd} (g cm^{-3})	8, 1.567
diffractometer	Bruker APEX-II CCD
μ (cm^{-1})	0.000
radiation type	electron
radiation wavelength (Å)	0.02508
F(000)	914
goodness of fit	1.486
temperature (K)	293(2)
number of reflections collected/unique	5027/2817
R_{int}	0.1795
R_1 ($I > 2.00\sigma(I)$)[a]	0.1987
wR_2 ($I > 2.00\sigma(I)$)[b]	0.4509
CCDC deposition number	2236267

[a] $R_1 = \Sigma||F_o|-|F_c||/\Sigma|F_o|$, [b] $wR_2 = [\Sigma w|F_o^2-F_c^2|^2/\Sigma w(F_o^2)^2]^{1/2}$

Section 2: Structures and physicochemical properties of CO_2 and C_2H_2

Supplementary Table 3. Structures and physicochemical properties of CO_2 and C_2H_2[21–23].

	CO_2	C_2H_2
	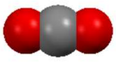	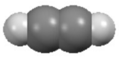
kinetic diameter (Å)	3.3	3.3
molecular size (Å^3)	3.2 × 3.3 × 5.4	3.3 × 3.3 × 5.7
MIN-2 size (Å)	3.339	3.340
standard boiling point (K)	216.55	188.40
critical temperature (K)	304.12	308.30
critical pressure (bar)	73.74	61.14
critical volume (m^3 kg^{-1})	94.07	112.20
polarizability (×10^{25} cm^3)	29.11	33.3
quadrupole moment (×10^{-40} cm^2)	–13.4	20.5

Section 3: Calculation of IAST selectivity

Supplementary Table 4. The dual-site Langmuir-Freundlich fitting parameters of CO_2 sorption data at different temperatures.

	200 K	220 K	240 K	260 K	280 K	300 K	320 K	340 K	360 K
n_{m1}	0.31095	0.25254	0.54276	0.29568	0.29568	1.12368	4.7692	0.36883	0.29707
n_{m2}	2.39602	5.90028	3.17592	1.66608	1.66608	1.12368	4.79664	0.36883	0.29707
b_1	1.21512	0.98529	1.99E-6	1.864E-7	1.86E-7	0.00931	6.62E-4	0.01091	0.00661
b_2	0.00226	0.0039	0.02641	0.02222	0.02222	0.00931	6.62E-4	0.01091	0.00661
t_1	1.15931	1.04777	0.31455	0.26451	0.26451	1.0942	0.80539	0.93055	0.96219
t_2	0.81998	0.99113	1.33953	1.10487	1.10487	1.0942	0.80539	0.93055	0.96219
R^2	0.9996	0.9996	0.9998	0.9999	0.9998	0.9998	0.9983	0.9980	0.9979

Supplementary Table 5. The dual-site Langmuir-Freundlich fitting parameters of C_2H_2 sorption data at different temperatures.

	200 K	220 K	240 K	260 K	280 K	300 K	320 K	340 K	360 K
n_{m1}	12.93665	0.45545	0.32295	35.50842	52.88381	58.22708	4.7692	2.94161	1.36217
n_{m2}	0.25777	0.3187	16.55781	0.31837	0.3599	0.13565	4.79664	2.94161	1.36217
b_1	0.00442	2.18E-4	2.39E-4	0.00121	6.03E-4	3.45E-4	6.62E-4	0.00182	0.00365
b_2	2.33762E-5	0.18689	0.00403	7.54E-6	1.61E-6	1.73E-12	6.62E-4	0.00182	0.00365
t_1	2.44582	0.49301	0.49949	1.70374	1.4386	1.1384	0.80539	0.9224	0.95457
t_2	0.42166	1.72121	2.29703	0.36048	0.31937	0.15287	0.80539	0.9224	0.95457
R^2	0.9989	0.9997	0.9994	0.9997	0.9995	0.9996	0.9983	0.9994	0.9991

Section 4: Solid-state NMR

Supplementary Table 6. Tentative assignments of ^{13}C NMR of FDC–3a and OPTz-t3da ligand.

<table>
<tr><th colspan="2" rowspan="2">Carbon numbering</th><th>FDC–3a</th><th rowspan="2">Carbon numbering</th><th colspan="2">OPTz-t3da</th></tr>
<tr><th>ssNMR
δ (ppm)</th><th>liquid
NMR
δ (ppm)</th><th>ACD NMR
prediction
δ (ppm)</th></tr>
<tr><td rowspan="7">quaternary
carbon</td><td>C1, C1'</td><td>177.2, 171.5</td><td>C1 (×2)</td><td>167.7</td><td>170.6</td></tr>
<tr><td>C11</td><td>147.8</td><td>C11</td><td>140.4</td><td>144.9</td></tr>
<tr><td>C8, C8'</td><td>143.5, 138.3</td><td>C8 (×2)</td><td>143.9</td><td>142.0</td></tr>
<tr><td>C6, C6'</td><td>139.9, 138.3</td><td>C12 (×2)</td><td rowspan="2">140.6
139.3</td><td>140.0</td></tr>
<tr><td>C12, C12'</td><td>136.6 (×2)</td><td>C6 (×2)</td><td>140.0</td></tr>
<tr><td>C17, C17'</td><td rowspan="2">135.2 (×2)</td><td>C2 (×2)</td><td>132.2</td><td>130.6</td></tr>
<tr><td>C2, C2'</td><td>C17 (×2)</td><td>122.7</td><td>125.3</td></tr>
<tr><td rowspan="10">tertiary
carbon</td><td>C14, C14'</td><td rowspan="5">132.7, 132.3
130.3 (×3)
123.7, 123.4
121.8, 121.3</td><td>C14 (×2)</td><td>134.0</td><td>134.6</td></tr>
<tr><td>C9</td><td>C5 (×2)</td><td>132.1</td><td>130.4</td></tr>
<tr><td>C16, C16'</td><td>C3 (×2)</td><td>129.9</td><td>130.4</td></tr>
<tr><td>C15, C15'</td><td>C10 (×2)</td><td>129.6</td><td>129.5</td></tr>
<tr><td>C13, C13'</td><td>C7 (×2)</td><td>128.4</td><td>128.1</td></tr>
<tr><td>C5, C5'</td><td rowspan="4">134.3
129.6
128.9
128.2
127.7
127.1
126.1
125.0</td><td>C9</td><td>126.9</td><td>127.1</td></tr>
<tr><td>C3, C3'</td><td>C16 (×2)</td><td>128.3</td><td>125.9</td></tr>
<tr><td>C10, C10'</td><td>C4 (×2)</td><td rowspan="2">122.6
123.3</td><td>125.4</td></tr>
<tr><td>C7, C7'</td><td>C15 (×2)</td><td>124.1</td></tr>
<tr><td>C4, C4'</td><td>116.6, 115.4</td><td>C13 (×2)</td><td>118.0</td><td>120.3</td></tr>
</table>

Section 5: Theoretical calculations

Supplementary Table 7. Theoretically optimized unit-cell parameters of FDC–3a at different phases.

Phase	*a* (Å)	*b* (Å)	*c* (Å)	*V* (Å^3)	E_{ads} (kJ mol^{-1})
activated	21.40<br>(21.18)	11.04<br>(11.18)	22.16<br>(21.91)	5188.1<br>(5188.1)	–
CO_2-adsorbed	21.40	11.04	22.16	5188.1	-26.5
C_2H_2-adsorbed	21.40	11.04	22.16	5188.1	-40.4

[a]Experimental values are shown in parentheses.

Supplementary References

1. Sheldrick, G. M. A short history of SHELX. *Acta Crystallogr. Sect. A: Found. Crystallogr.* **64**, 112–122 (2008).

2. Nishibori, E. *et al.* The large Debye–Scherrer camera installed at SPring-8 BL02B2 for charge density studies. *Nucl. Instrum. Methods Phys. Res. A* **467**/**468**, 1045–1048 (2001).

3. Takata, M. *et al.* High resolution Debye-Scherrer camera installed at SPring-8. *Advances in X-Ray Analysis* **45**, 377–384 (2002).

4. Xie, Y. *et al.* Tuning the topology of three-dimensional covalent organic frameworks via steric control: from pts to unprecedented ljh. *J. Am. Chem. Soc.* **143**, 7279–7284 (2021).

5. Kärger, J., Ruthven, D. M. & Theodorou, D. N. *Diffusion in nanoporous materials* (WILEY-VCH Verlag GmbH & Co. kGaA, 2012).

6. van Gorkom, L. C. M., Hook, J. M., Logan, M. B., Hanna, J. V. & Wasylishen, R. E. Solid-state lead-207 NMR of lead(II) nitrate: localized heating effects at high magic angle spinning speeds. *Magn. Reson. Chem.* **33**, 791–795 (1995).

7. Hohenberg, P. & Kohn, W. Inhomogeneous electron gas. *Phys. Rev. B* **136**, B864–B871 (1964).

8. Kohn, W. & Sham, L. J. Self-consistent equations including exchange and correlation effects. *Phys. Rev.* **140**, 1133–1138 (1965).

9. Clark, S. J. *et al.* First principles methods using CASTEP. *Z. Kristallogr.* **220**, 567–570 (2005).

10. Perdew, J. P., Burke, K. & Ernzerhof, M. Generalized gradient approximation made simple. *Phys. Rev. Lett.* **77**, 3865–3868 (1996).

11. Grimme, S. Accurate description of van der Waals complexes by density functional theory including empirical corrections. *J. Comput. Chem.* **25**, 1463–1473 (2004).

12. Grimme, S. Semiempirical GGA-type density functional constructed with a long-range dispersion correction. *J. Comput. Chem.* **27**, 1787–1799 (2006).

13. Metropolis, N., Rosenbluth, A. W., Rosenbluth, M. N., Teller, A. H. & Teller, E. Equation of state calculations by fast computing machines. *J. Chem. Phys.* **21**, 1087–1092 (1953).

14. Rappe, A. K., Casewit, C. J., Colwell, K. S., Goddard, W. A. & Skiff, W. M. Uff, a full periodic-table force-field for molecular mechanics and molecular-dynamics simulations. *J. Am. Chem. Soc.* **114**, 10024–10035 (1992).

15. Casewit, C. J., Colwell, K. S. & Rappe, A. K. Application of a universal force field to organic molecules. *J. Am. Chem. Soc.* **114**, 10035–10046 (1992).

16. Casewit, C. J., Colwell, K. S. & Rappe, A. K. Application of a universal force field to main group compounds. *J. Am. Chem. Soc.* **114**, 10046–10053 (1992).

17. M. J. Frisch, *et al. Gaussian 09*, revision D.01; Gaussian, Inc: Wallingford, CT, 2013.

18. Grimme, S., Antony, J., Ehrlich, S., Krieg, H., A consistent and accurate *ab initio* parametrization of density functional dispersion correction (DFT-D) for the 94 elements H-Pu. *J. Chem. Phys.* **132**, 154104 (2010).

19. Gu, C. *et al.* Design and control of gas diffusion process in a nanoporous soft crystal. *Science* **363**, 387–391 (2019).

20. Su, Y., Otake, K.-i., Zheng, J.-J., Horike, S., Kitagawa, S. & Gu, C. Separating water isotopologues using diffusion-regulatory porous materials. *Nature* **611**, 289–294 (2022).

21. Li, J.-R., Kuppler, R. J. & Zhou, H.-C. Selective gas adsorption and separation in metal-organic frameworks. *Chem. Soc. Rev.* **38**, 1477–1504 (2009).

22. Webster, C. E., Drago, R. S. & Zerner, M. C. Molecular dimensions for adsorptives. *J. Am. Chem. Soc.* **120**, 5509–5516 (1998).
23. Sing, K. S. W. & Williams, R. T. The use of molecular probes for the characterization of nanoporous adsorbents. *Part. Part. Syst. Charact.* **21**, 71–79 (2004).
