# Supplementary figures and images for: Switching molecular recognition selectivities by temperature in a diffusion-regulatory porous material
Source: Nat Commun. 2024 Jan 2;15:144. doi: 10.1038/s41467-023-44424-3 (PMC10761840; doi:10.1038/s41467-023-44424-3)

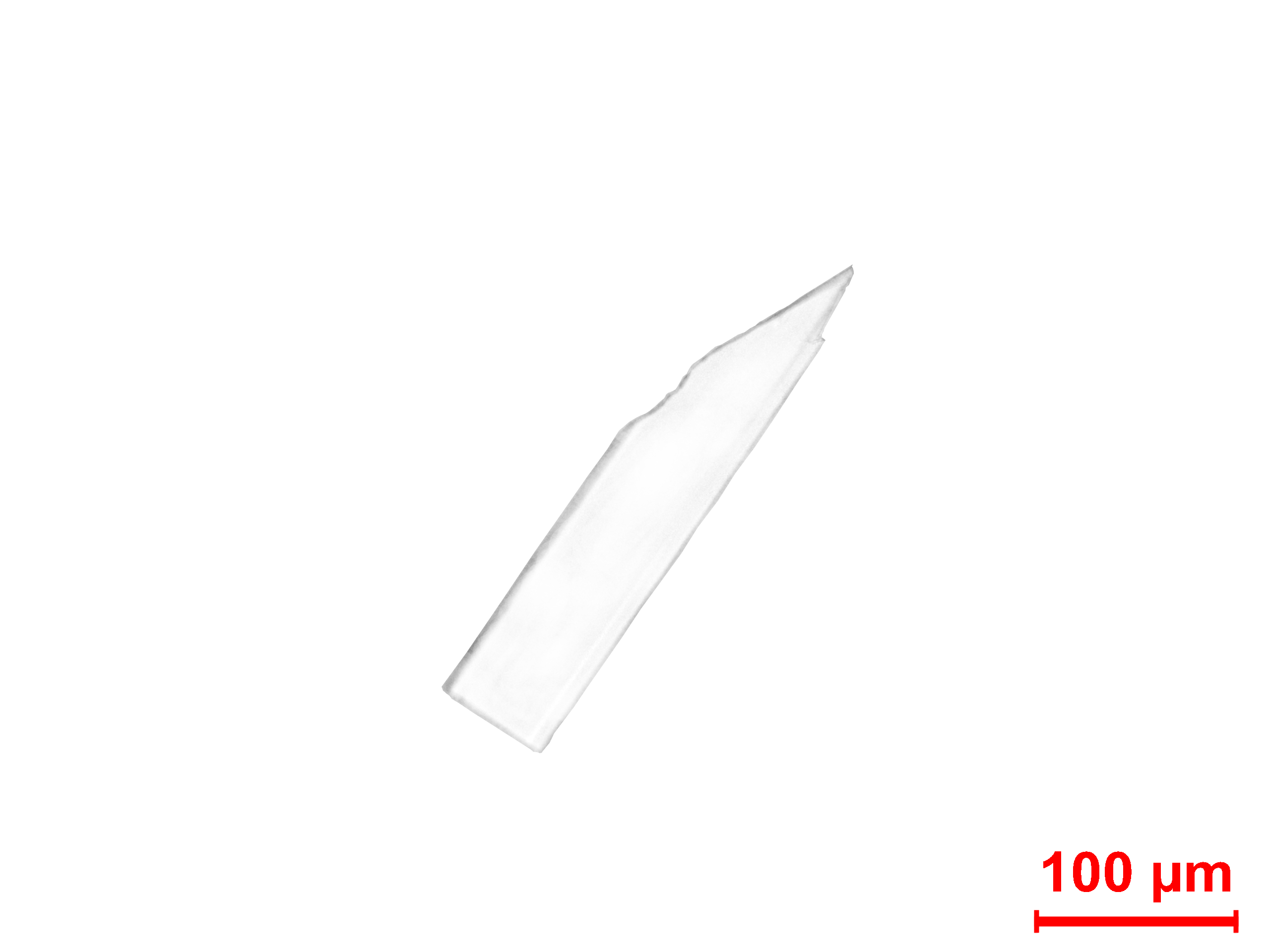

Supplement: Supplementary file 3 — source data [file 41467_2023_44424_MOESM3_ESM.zip › Figure S2.png]

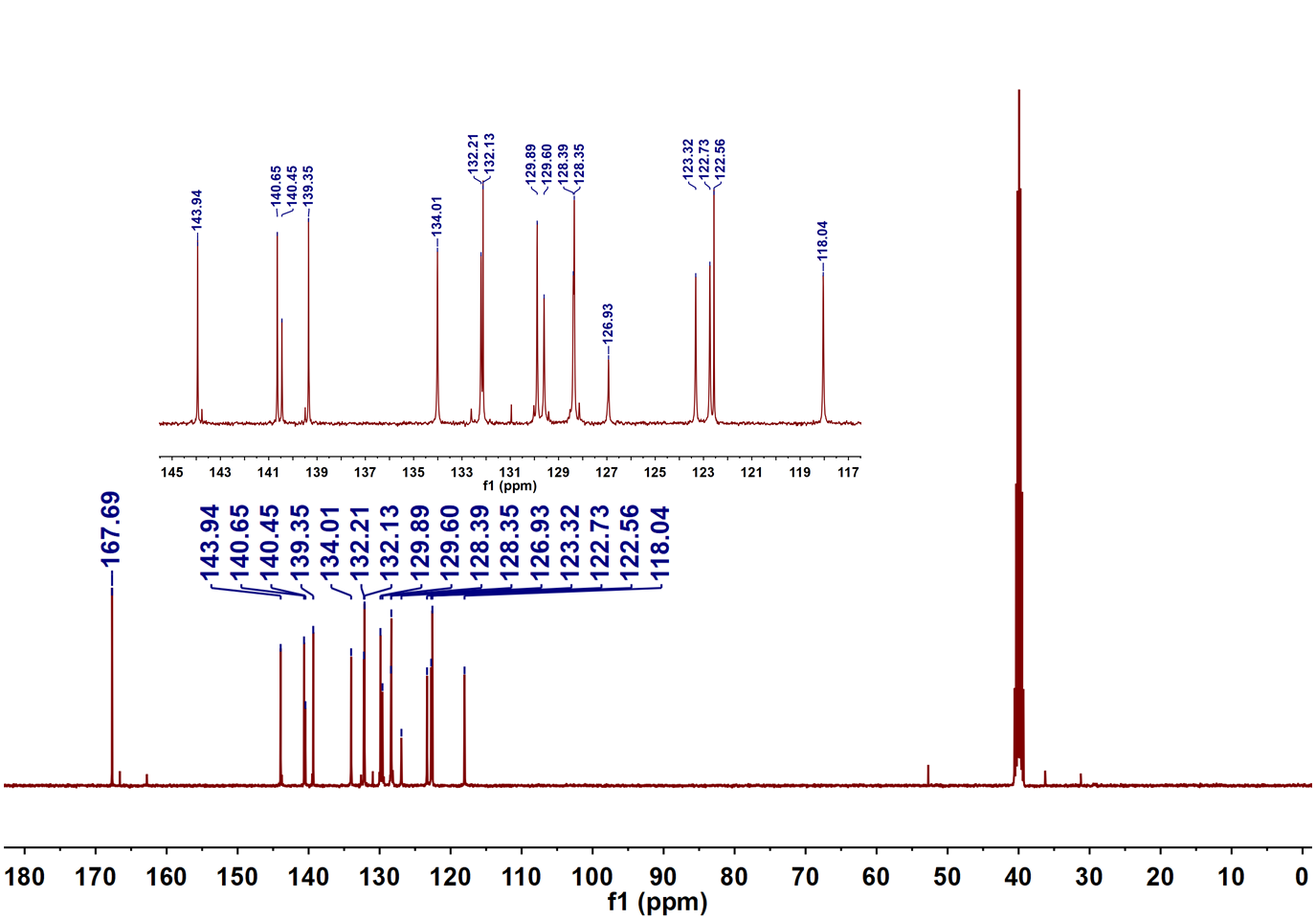

Supplement: Supplementary file 3 — source data [file 41467_2023_44424_MOESM3_ESM.zip › Figure S25.png]
